# OrgaCCC: Orthogonal graph autoencoders for constructing cell-cell communication networks on spatial transcriptomics data

**DOI:** 10.1371/journal.pcbi.1013212

**Published:** 2025-06-27

**Authors:** Xixuan Feng, Shuqin Zhang, Limin Li

**Affiliations:** 1 School of Mathematics and Statistics, Xi’an Jiaotong University, Shaanxi, China; 2 School of Mathematical Sciences, Fudan University, Shanghai, China; University of Pittsburgh, UNITED STATES OF AMERICA

## Abstract

Cell-cell communication (CCC) is a fundamental biological process essential for maintaining the functionality of multicellular organisms. It allows cells to coordinate their activities, sustain tissue homeostasis, and adapt to environmental changes. However, understanding the mechanisms underlying intercellular communication remains challenging. The rapid advancements in spatial transcriptomics (ST) have enabled the analysis of CCC within its spatial context. Despite the development of several computational methods for inferring CCCs from ST data, most rely on literature-curated gene or protein interaction lists, which are often inadequate due to the restricted gene coverage. In this work, we propose OrgaCCC, an orthogonal graph autoencoders approach for cell-cell communication inference based on deep generative models. OrgaCCC leverages the information of gene expression profiles, spatial locations and ligand-receptor relationships. It captures both cell/spot and gene features using two orthogonally coupled variational graph autoencoders across cell/spot and gene dimensions and combines them by maximizing the similarity between their reconstructed cell/spot features. Numerical experiments on five ST datasets demonstrate the superiority of OrgaCCC compared with state-of-the-art methods in CCC inference at the cell-type level, cell/spot level, and ligand-receptor level, in terms of inference accuracy and reliability.

## Author summary

Cell-cell communication is vital for tissue function and homeostasis, but understanding its mechanisms is challenging due to the complexity of interactions. While spatial transcriptomics (ST) offers a powerful approach to study CCC in its spatial context, existing computational methods often rely on limited gene interaction data, failing to capture the full complexity of spatial and cellular relationships. We introduce OrgaCCC, a deep generative model designed to infer cell-cell communication from spatial transcriptomics (ST) data using orthogonal graph autoencoders. The model employs two distinct autoencoders operating at the cell/spot and gene levels, respectively. These autoencoders integrate gene expression, spatial localization, and ligand-receptor interactions to capture both cellular and molecular features. By maximizing the similarity between reconstructed cell/spot features, OrgaCCC improves the accuracy and reliability of CCC inference, offering a more precise framework for identifying intercellular interactions in spatially resolved tissues.

## Introduction

Cell-cell communication (CCC) refers to the transmission of signals between cells through various signaling molecules, including cytokines, membrane proteins, and ligand-receptor pairs. This process is essential for coordinating physiological functions such as development, immune responses, and tissue repair, ensuring the efficient, precise, and orderly operation of multicellular organisms [1]. The mechanisms of CCC are diverse, primarily involving autocrine, paracrine, endocrine, and juxtacrine signaling. Among these, ligand-receptor-mediated intercellular signaling plays a particularly pivotal role. When ligands bind to receptors on the cell surface, the signals are transmitted intracellularly, triggering a cascade of downstream pathways that regulate fundamental cellular activities such as proliferation, differentiation, migration, and apoptosis. Thus, CCC is a critical component of the intricate regulatory networks that govern organismal function. Studying CCC not only enhances our understanding of the fundamental mechanisms of life but also provides valuable insights for developing novel strategies in disease prevention, treatment, and therapeutic discovery.

With the advancement of single-cell RNA sequencing (scRNA-seq) technology, several computational methods have been designed to estimate CCC activities based on scRNA-seq data and ligand-receptor databases [2–4]. Examples include iTALK [5], CellPhoneDB [2], CellChat [3], and others. iTALK [5] identifies highly or differentially expressed genes and matches them with the ligand-receptor database to pinpoint active communication pathways between different cell types. CellPhoneDB [2] considers the subunit architecture of ligands and receptors to accurately represent heteromeric complexes and evaluates the significance of cellular interactions between two cell types using permutation tests. CellChat [3] models the intercellular interactions through the mass action model, incorporating differentially over-expressed ligands and receptors for each cell group, and infers statistically and biologically significant CCCs by permutation tests. NicheNet [6] predicts ligand–target links between interacting cells by integrating expression data with prior knowledge of ligand–receptor interactions, signal transduction and gene regulatory networks. CrossChat [7] identifies global and local cellular communication hierarchical structures based on ligand–receptor expression patterns, enabling flexible analysis of complex tissue-level interactions. While these tools effectively infer CCCs from scRNA-seq data and ligand-receptor databases, they do not incorporate spatial location information of cells. Since CCC can be influenced by spatial proximity, the absence of spatial context in these methods may lead to false positive results.

The rapid development of spatial transcriptomics (ST) technology has opened up new avenues for analyzing CCC within its spatial context. ST technology varies from spot-based capture techniques to high-resolution imaging methods. Spot-based ST technology provides near-cellular resolution gene expressions while single-cell ST (scST) technology generates gene expression profiles at single-cell resolution, both preserving their spatial positions [8, 9]. These techniques significantly enhances the accuracy of CCC predictions. Computational methods developed for scST data include Tensor-cell2cell [10], CellPhoneDB v3, v5 [11, 12], CellChat v2 [13], NiCo [14], and so on. Tensor-cell2cell [10], an unsupervised method based on tensor decomposition, deciphers context-driven intercellular communication by capturing multiple phases, states, or locations of cells simultaneously. CellPhoneDB v3 [11] builds upon the original CellPhoneDB by incorporating spatial information, restricting CCC analysis to cells within the same microenvironment as defined by their spatial context. CellPhoneDB v5 [12] further improves the framework by introducing spatial context-aware scoring for spatial transcriptomics data, enhancing confidence estimation for inferred interactions, and providing more advanced visualization tools. CellChat v2 [13] improves the original version by integrating spatial information and implementing a multi-layer network model, making it suitable for more complex single-cell and spatial transcriptomics analyses. NiCo [14] combines spatial transcriptomics with niche covariation analysis to uncover dynamic interactions between cells and their microenvironment, revealing spatial dependencies and underlying regulatory mechanisms of cell-cell communication. Although these methods leverage single-cell spatial resolution to improve CCC inference, they are restricted to scST data and are not applicable to spot-based spatial transcriptomics datasets. In addition, their reliance on predefined ligand–receptor databases makes them sensitive to sparse gene coverage or missing ligand–receptor pairs, which can compromise prediction accuracy and interpretability.

In parallel, a number of CCC inference methods have been proposed for both single-cell and spot-based ST data, potentially alleviating the above limitation of data type restriction. Giotto [15] integrates known ligand-receptor data from existing databases and estimates interactions between two cell types by calculating the spatial co-expression of ligand-receptor gene pairs in neighboring cells. COMMOT [16] incorporates spatial distance constraints into CCC inference and uses optimal transport to simultaneously infer CCCs across a large number of ligand-receptor pairs. SpatialDM [17] leverages spatial autocorrelation, using Moran’s I and permutation test to identify statistically significant ligand–receptor interactions based on co-expression patterns. However, like the previous methods, they still depend on curated ligand-receptor databases, where the challenge of sparse gene coverage remains.

In contrast, some existing methods take into account the entire gene expression profiles to predict CCCs. For instance, DeepLinc [18] generates the cell-cell interaction network with all genes using variational graph autoencoders and incorporates the spatial locations of cells. Spacia [19] is a multiple-instance learning-based computational framework that models the response of individual receiver cells to their spatially proximal sender cells, enabling the inference of cell–cell communication at single-cell resolution from spatial transcriptomics data. However, these approaches do not account for ligand-receptor interactions, limiting their ability to capture biologically relevant communication pathways.

To address the challenges mentioned above, we propose OrgaCCC, an orthogonal graph autoencoders approach for cell-cell communication (CCC) inference based on deep generative models. OrgaCCC incorporates both the spatial location information of cells/spots and known ligand-receptor relationships. It captures both cell/spot and gene features using two separate variational graph autoencoders, which are integrated by maximizing the similarity between their reconstructed gene expression matrix. The final reconstructed cell-cell relationship matrix represents the inferred CCC network. We validated OrgaCCC by conducting experiments on five spatial transcriptomics datasets and comparing the results with state-of-the-art CCC inference methods. The results demonstrate the advantages of OrgaCCC at the cell-type level, cell/spot level, and ligand-receptor level. Furthermore, through robustness analysis, we showed that OrgaCCC can make stable predictions even from incomplete or noisy cell profiles, outperforming other methods in terms of reliability and accuracy.

## Results

### Overview of OrgaCCC

Given the gene expression matrix $X\in {\mathbb{R}}^{m\times n}$ corresponding to *m* genes and *n* cells/spots. Orthogonal graph autoencoders (OrgaCCC) infer cell-cell communication through two orthogonal graph autoencoders operating at the cell/spot level and gene level. These autoencoders leverage the gene expression profiles of individual cells/spots, their spatial locations, and known ligand-receptor relationships, under the assumption that neighboring cells/spots are more likely to interact than those randomly selected. At the cell/spot level, a neighboring cell/spot-level graph is constructed and represented as an adjacency matrix *A*_*c*_, where nodes represent individual cells/spots and edges encode their spatial relationships based on physical proximity, derived from spatial transcriptomics data. This graph provides a spatial perspective on physical cell-cell interactions, offering an incomplete and potentially noisy approximation of the full cell-cell communication network. The cell/spot-level Variational Graph Autoencoder (VGAE) aims to reconstruct both the gene expression profiles ${\hat{X}}_{c}$ and the cell-cell interaction network ${\hat{A}}_{c}$, capturing variations in expression patterns across cells/spots. The resulting network reflects the CCCs among distinct cells/spots. At the gene level, a gene-gene graph is constructed as an adjacency matrix *A*_*g*_, where nodes correspond to individual genes and edges represent known ligand-receptor interactions. The gene-level VGAE similarly reconstructs both the gene expression profiles ${\hat{X}}_{g}$ and the ligand-receptor interaction network ${\hat{A}}_{g}$, capturing variations in expression patterns across genes. The inferred network captures predicted ligand-receptor interactions. These two autoencoders are integrated by maximizing the cosine similarity between the reconstructed gene expression matrices, ensuring joint model training and a consistent distribution of the lower-dimensional representations of gene expression. By leveraging graph-based representations in both cell/spot and gene dimensions through orthogonal graph autoencoders, OrgaCCC simultaneously captures cell/spot-level and gene-level features. This approach preserves local microenvironments, reflects ligand-receptor interactions, and generates a comprehensive landscape of the cell-cell communication network.

OrgaCCC was applied to five spatial transcriptomics datasets shown in Table 1. We performed a comprehensive comparative analysis of OrgaCCC against several other methods, including CellChat [13], NiCo [14], iTALK [5], and COMMOT [16] for the predicted cell-cell communications at the cell-type level, cell level and ligand-receptor level. Note that for the method DeepLinc [18], since it does not integrate information from ligand-receptor databases, and the final results are presented as communication probabilities between each pair of cell types, our comparative analysis with DeepLinc was conducted solely at the cell level. For OrgaCCC, we also conducted downstream analysis on spectral clustering of the reconstructed cell/spot-level graph ${\hat{A}}_{c}$, where the clusters exhibit more accurate representation of cell communication and interactions, as well as the developmental trajectories of the cells. We additionally identified key genes involved in the cell-cell communications, selecting a subset of top-ranked genes for biological enrichment analysis. This helped us gain insights into the functional roles and mechanisms of these genes in biological processes. Furthermore, we conducted a series of robustness tests, demonstrating that OrgaCCC maintains high prediction accuracy and stability even with incomplete or noisy data. These results highlight OrgaCCC’s robustness and reliability in CCC inference from complex and imperfect datasets.

**Fig 1:**
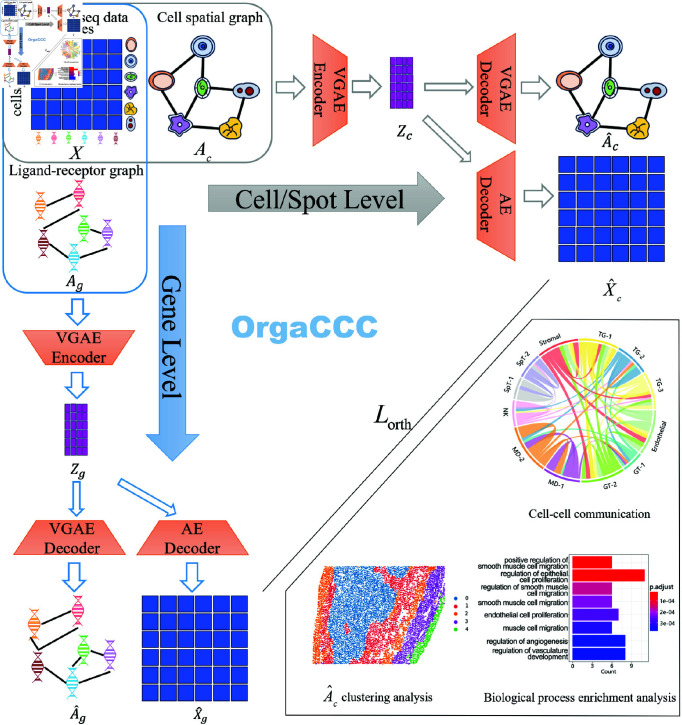
Overview of the OrgaCCC. The model inputs a gene expression matrix, a cell-cell/spot-spot graph constructed from spatial distances, and a ligand-receptor gene relationship graph. It consists of two orthogonal graph autoencoders. Gene-level autoencoder (vertical process): the gene-level graph ${𝒢}_{g}$ is encoded through the variational graph autoencoder (VGAE), and then decoded to reconstruct the gene-gene relationship graph and the gene expression matrix. Cell/Spot-level autoencoder (horizontal process): the cell/spot-level graph ${𝒢}_{c}$ is encoded and decoded using VGAE to reconstruct the cell-cell/spot-spot relationship graph and gene expression matrix. These two VGAEs are integrated via the cross-modal loss function $L_{\text{orth}}$, enabling joint training. The lower right corner shows the visualization of the cellular communication network (chordal plot) and some of the downstream analysis, e.g. visualization of reconstructed cell/spot-level graph clustering, bioprocess enrichment analysis of the significant genes involved in CCC activity.

**Table 1:** Dataset Information.

Platform	Cell/Spot number	Gene number	Tissue
STARmap	7224	903	mouse placenta [22]
MERFISH	4975	160	mouse hypothalamic preoptic region [23]
seqFISH+	523	10000	mouse secondary somatosensory cortex [24]
Visium	1037	648	mouse cortex [25]
Visium	2649	15882	human intestine [26]

### CCC results on the STARmap data of mouse placenta

**Fig 2:**
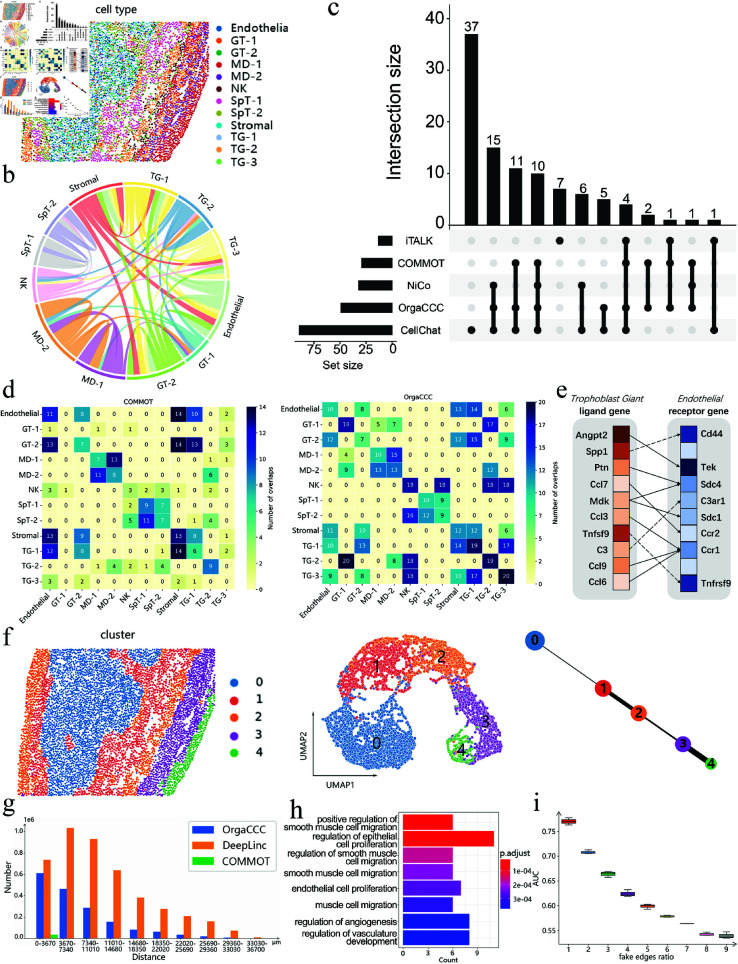
Analysis of cell-cell communication results on the STARmap data of the mouse placenta. **a**. Spatial distribution of the cell types. **b**. Chord plot of cell-cell communications identified using OrgaCCC. Each color represents a specific cell type, the color of the arc indicates the sender of the signal, and the width of the arc reflects the strength of the communication. GT: Glandular Trophoblast; MD: Maternal Decidua; NK: natural killer; SpT: Spongiotrophoblast; TG: Trophoblast Giant. **c**. Comparison of cell-type-level CCCs predicted by OrgaCCC, CellChat, COMMOT, NiCo, iTALK. Numbers count the cell-type-level communication pairs. **d**. Number of overlapping ligand-receptor pairs predicted by COMMOT (left) with CellChat, NiCo, iTALK, and OrgaCCC, and predicted by OrgaCCC (right) with CellChat, NiCo, iTALK, and COMMOT. **e**. Predicted ligand-receptor gene pairs mediating communication between trophoblast giant and endothelial using OrgaCCC. The colors from darkest to lightest represent the rankings from top to bottom. The solid line indicates that the ligand-receptor pair is also recognized by one of the other methods (CellChat, COMMOT, NiCo, or iTALK). The dashed line indicates that the pair was only recognized by OrgaCCC. **f**. The cell clusters obtained by spectral clustering on the reconstructed cell-level graph ${\hat{A}}_{c}$, and the corresponding UMAP and PAGA graphs. **g**. Number of the communicating cell pairs at different distances predicted by OrgaCCC, DeepLinc and COMMOT. **h**. Enrichment analysis for the top-ranked significant genes in CCCs. **i**. Robustness of OrgaCCC when a different number (1-9 times the size of *A*_*c*_) of fake edges are added to the cell spatial graph *A*_*c*_.

We first analyzed the STARmap data of mouse placenta, which comprises 7,224 cells and 903 genes. Fig 2a depicts the spatial distribution of the cell types, and Fig 2b illustrates the cell-type-level communications identified using OrgaCCC. Endothelial cells exhibited significant communication with glandular trophoblast (GT-2), stromal cells, and trophoblast giant (TG-1). Glandular trophoblast (GT-1) was observed to interact with maternal decidual cells (MD-2) and trophoblast giant (TG-2), contributing to the maintenance of placental development. Spongiotrophoblast (SpT) and natural killer (NK) cells demonstrated strong communication patterns. These interactions reveal the intricate relationships among different cell types. For example, TG-endothelial interactions anchor TG cells to the vessel lumen, ensuring proper blood flow and placental development [27]. Stromal cells release growth factors and cytokines to regulate the proliferation and migration of endothelial cells, while endothelial cells secrete signaling molecules to modulate stromal cell functions [28]. In addition, MD, GT, and TG cells communicate through hormones, cytokines, and chemokines to coordinate placental growth and function [29].

The cell-type-level CCCs predicted by OrgaCCC, CellChat [13], COMMOT [16], NiCo [14] and iTALK [5] are then compared. Fig 2c summarizes the comparative results. OrgaCCC identified a total of 50 pairs of cell-type-level communications, all of which overlapped with predictions from other methods. In contrast, CellChat predicted a larger total of 89 cell-type-level communications, while 58.4% overlapped with those from other methods. COMMOT and NiCo yielded significantly fewer predictions (29 and 32 pairs, respectively), though all their identified pairs aligned with consensus results from other methods. iTALK predicted 13 cell-type-level communications, while 46.2% overlapped with those from other methods. These findings show the high consistency of OrgaCCC’s predictions with other methods, highlighting OrgaCCC’s reliability in predicting intercellular communications.

To assess the functional importance of specific ligand-receptor pairs in mediating intercellular communication, we performed a systematic perturbation analysis. For each ligand-receptor pair of interest between two cell types, we inferred the CCC network by removing the corresponding edge from the complete ligand-receptor interaction graph. The impact of each ligand-receptor pair was then quantified by calculating the edge reduction ratio: the proportion of predicted CCCs lost when using the perturbed network relative to the original complete network. This ratio serves as a quantitative measure of the ligand-receptor pair’s influence on intercellular communication, where higher values indicate more critical roles in maintaining communication between the corresponding cell types. For each cell type pair, we ranked the impact of all ligand-receptor gene pairs and selected the top 20 as the key pairs responsible for mediating intercellular communication. The identified ligand-receptor pairs by OrgaCCC were compared with those obtained using CellChat, COMMOT, NiCo and iTALK. Specifically, we examined the overlap between the ligand-receptor gene pairs predicted by each method and the union set predicted by the other four methods. Fig 2d illustrates the results for COMMOT and OrgaCCC, while the results for CellChat, NiCo and iTALK are reported in the supplementary materials (S1 Fig d). OrgaCCC not only demonstrates a higher overlap with the other methods, but also identifies meaningful ligand-receptor gene pairs that are not detected by others. For example, Fig 2e highlights the top ten ligand-receptor pairs identified by OrgaCCC as mediators of communication between TG cells and endothelial cells. The *Spp1-Cd44* pairing supports normal placental development and function by promoting cell adhesion, migration, immunomodulation and angiogenesis [30, 31]. This pairing plays a key role in regulating placental angiogenesis and maintaining an appropriate immune environment.

To explore the patterns inherent in the cell-cell communication network predicted by OrgaCCC, we applied spectral clustering to the final cell-level graph ${\hat{A}}_{c}$. Five subcellular clusters were identified (Fig 2f). Different from the original cell type clusters shown in Fig 2a, these clusters better reflect the intercellular communication relationships and the developmental trajectory of tissues from internal to external regions. The cell types contained in each cell class are more likely to communicate with one another. For example, cluster 2 primarily includes three cell types: TG, NK, and GT (S1 Fig c). As shown in Fig 2b, these three cell types exhibit strong cell communication relationships. To further analyze the developmental trajectories at the tissue level, we visualized the latent embeddings from spectral clustering using UMAP. Additionally, to elucidate cell state transitions and differentiation pathways, we applied partition-based graph abstraction (PAGA), which provides a clear visualization of the relationships between the cell clusters (Fig 2f). For instance, cells originating from cluster 0 (comprising TG, endothelial, GT, and stromal cells) represent the initial stages of early placental development and angiogenesis. These cells then progress to cluster 1, which is dominated by SpT cells, indicating further differentiation of TG cells [32]. Finally, the developmental endpoint is reached at cluster 4, which is primarily composed of MD and GT cells. This cluster marks the close integration between the placenta and maternal decidua, promoting placental maturation and functional stabilization [33, 34].

Cells in close proximity are often supposed to be more likely to engage in cell-cell communications. To test this hypothesis, we analyzed the predicted CCCs at the cellular level, expecting that the number of predicted interacting cell pairs would increase as the distance between cells decreases. We compared the predictions generated by OrgaCCC, COMMOT and DeepLinc [18]. For COMMOT, we followed the official tutorial and set a distance threshold of 500 μm (Fig 2g). OrgaCCC’s predictions align well with the assumption that shorter distances correlate with higher frequencies of communications, while it also captures instances of long-range CCC. This demonstrates that OrgaCCC not only effectively identifies spatially dependent interactions but also accounts for communication events across greater distances.

To pinpoint specific gene expressions that play crucial roles in cell-cell communications, we employed the sensitivity analysis approach (see Methods). We calculated gene sensitivity to identify key expressed genes and selected the top 8% for bioenrichment analysis. The results revealed that these genes are predominantly involved in critical biological processes (Fig 2h). For example, the positive regulation of smooth muscle cell migration in the mouse placenta promotes blood vessel formation and remodeling, which is crucial for nutrient and gas exchange between the mother and fetus. By enhancing smooth muscle cell migration, this process stabilizes and remodels the vascular wall in the placenta, thereby supporting fetal growth and development [35]. Additionally, the regulation of epithelial cell proliferation plays a vital role in placental function, particularly in the proliferation of TG cells, which is a major cell type in the placenta. Proper regulation of TG cell proliferation ensures the structural integrity of the placenta and its ability to sustain healthy fetal development throughout pregnancy [36].

Finally, we evaluated the robustness of OrgaCCC using the model robustness analysis (see Methods). By randomly introducing fake edges between cell pairs in the original cell spatial graph *A*_*c*_, we trained the model with the perturbed *A*_*c*_, and predicted the CCCs. This process was repeated 30 times, and the AUCs comparing the CCC network predicted with the perturbed *A*_*c*_ and the original *A*_*c*_ are shown in Fig 2i. Even when the number of fake edges is three times of the number of cells, OrgaCCC can still efficiently filter out a substantial amount of noise and recover the cell-cell interactions. These results demonstrate that OrgaCCC exhibits strong tolerance and robust denoising capabilities for randomly introduced errors in cellular communication relations. Additionally, we tested the case when a certain percentage of edges were removed from *A*_*c*_ and the model was trained on the remaining network. The AUC values comparing the predicted graph obtained from training on the perturbed network with the original OrgaCCC predictions are shown in S1 Fig b. OrgaCCC successfully recovered the missing edges, further confirming its robustness and reliability.

### CCC results on the MERFISH data of the mouse hypothalamic preoptic region

**Fig 3:**
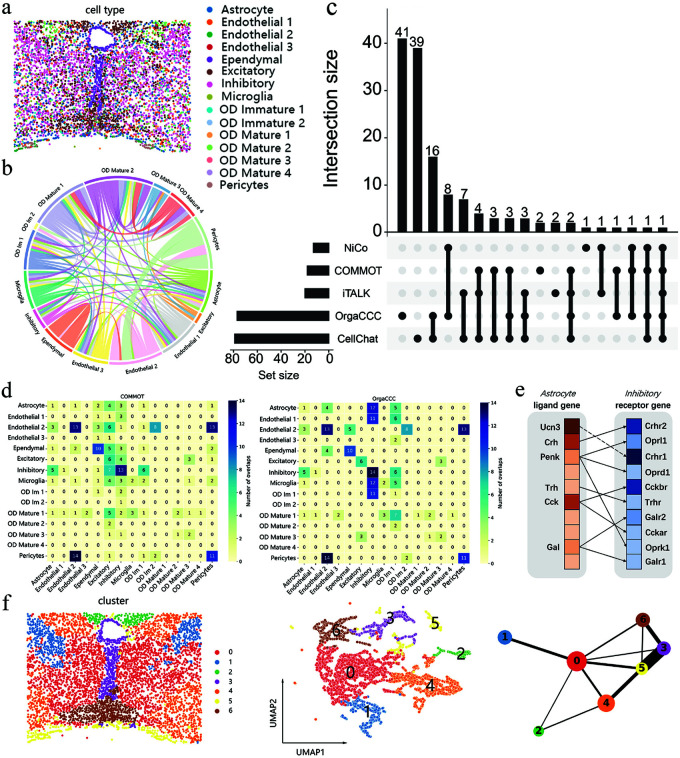
Analysis of cell-cell communication results on MERFISH data of mouse hypothalamic preoptic region. **a**. Spatial distribution of the cell types. **b**. Chord plot of cell-cell communications identified by OrgaCCC. Each color represents a different cell type, the color of the arc indicates the sender of the signal, and the width of the arc reflects the strength of the communication. **c**. Comparison of cell-type-level CCCs predicted by OrgaCCC, CellChat, COMMOT, NiCo and iTALK. Numbers count the cell-type-level communication pairs. **d**. Overlap of ligand-receptor pairs predicted by COMMOT (left) with CellChat, OrgaCCC, NiCo, and iTALK, as well as overlap predicted by OrgaCCC (right) with CellChat, iTALK, NiCo, and COMMOT. **e**. Predicted ligand-receptor gene pairs mediating communication between astrocyte giant and inhibitory neurons using OrgaCCC. The colors from darkest to lightest represent the rankings from top to bottom. The solid line indicates that the ligand-receptor pair is also recognized by one of the other methods (CellChat, COMMOT, NiCo, or iTALK). The dashed line indicates that the pair was only recognized by OrgaCCC. **f**. The cell clusters obtained by spectral clustering on the reconstructed cell-level graph ${\hat{A}}_{c}$, and the corresponding UMAP and PAGA graphs.

We next analyzed scST data of the mouse hypothalamic preoptic region generated using MERFISH. It consists of 5,338 cells and 160 genes. Fig 3a illustrates the spatial distribution of the cell types. The predicted cell-cell communications were categorized into distinct groups, as shown in Fig 3b. The first group mainly includes astrocytes, which interact extensively with multiple cell types, such as endothelial cells, inhibitory neurons, microglia, and oligodendrocytes (OD Im, OD Mature). The second group involves endothelial cells communicating with ependymal cells, inhibitory neurons, microglia, oligodendrocytes (OD Im, OD Mature), and pericytes. The third group highlights interactions between excitatory neurons, microglia, and oligodendrocytes (OD Im, OD Mature). Different cell types in each group collectively perform certain biological functions through cell-cell communications. For example, in the first group, astrocytes, together with endothelial cells, maintain the blood-brain barrier (BBB) by regulating its integrity through tight junctions [37]. They influence inhibitory neurons by releasing neurotransmitters (e.g., glutamate) and regulating extracellular ion concentrations (e.g., potassium) [38]. Astrocytes and microglia coordinate immune responses and neuroprotection in the central nervous system [39], while astrocyte-oligodendrocyte interactions support myelination, neuroregeneration, and immunomodulation [40].

The predicted cell-type-level intercellular communications of OrgaCCC were compared with CellChat, COMMOT, NiCo, and iTALK (Fig 3c). OrgaCCC predicted 77 interaction pairs, 46.7% of which overlapped with predictions from other methods. In contrast, CellChat, iTALK, COMMOT and NiCo predicted 79, 20, 18 and 13 interaction pairs, respectively, with overlap proportions of 50%, 90%, 89%, and 92%. OrgaCCC and CellChat inferred the largest number of overlapping interactions (16 pairs), and they also identified a number of unique communications that other methods missed. The key advantage of OrgaCCC lies in its ability not only to avoid sole reliance on traditional ligand-receptor databases, but also to integrate a data-driven approach capable of revealing cell-cell communications that may be missed by other methods.

We then compared the predicted responsible ligand-receptor pairs mediating the communications between each pair of different cell types, which are critical for maintaining the stability and functional integrity of the cell-cell communication network. Again we analyzed the results from OrgaCCC, CellChat, COMMOT, NiCo, and iTALK, focusing on the overlap between the ligand-receptor gene pairs predicted by each method and the union set predicted by the other four methods. Fig 3d illustrates the results of COMMOT and OrgaCCC, while the results for CellChat, NiCo and iTALK are provided in the supplementary materials (S2 Fig d). The ligand-receptor gene pairs predicted by OrgaCCC exhibit significant overlap with other methods. Moreover, OrgaCCC uniquely identified additional ligand-receptor pairs. For example, in the astrocyte-to-inhibitory neuron communication (Fig 3e), OrgaCCC uniquely predicts four ligand-receptor pairs: *Ucn3-Crhr1*, *Tac1-Tacr1*, *Tac2-Tacr3*, and *Ucn3-Crhr2*. The *Ucn3-Crhr1* pair plays a crucial role in regulating the stress response and anxiety by activating the hypothalamic-pituitary-adrenal (HPA) axis, affecting neuronal excitability and GABAergic activity in local circuits [41]. Similarly, the *Ucn3-Crhr2* pair is involved in chronic stress and metabolic regulation, contributing to behavioral adaptation and energy metabolism, with activation of *Crhr2* helping to cope with stress and maintain metabolic homeostasis [42, 43]. The *Tac1-Tacr1* pair, involved in pain transmission, stress response, and mood regulation, modulates anxiety and stress through the release of substance P and its interaction with *NK1R* [44]. Additionally, the *Tac2-Tacr3* pair regulates reproductive function by controlling gonadal hormone release, while also influencing stress responses and neural excitability [45]. These findings highlight OrgaCCC’s ability to uncover novel ligand-receptor interactions that may play critical roles in cell-cell communication.

We performed spectral clustering on the final cell-level graph ${\hat{A}}_{c}$, dividing it into seven subclusters. We then applied UMAP to reduce the dimensionality of the latent embeddings and constructed a PAGA plot to visualize the potential connections and structures among cell populations (Fig 3f). The newly delineated cell clusters reveal the intercellular communication relationships and clearly present the trajectory of the tissue. S2 Fig c illustrates the predominant cell types contained within each cluster. For example, cluster 4 primarily consists of inhibitory neurons, mature oligodendrocyte and astrocyte cell types. As shown in Fig 3b, these cell types exhibit significant intercellular communication relationships. In the PAGA plot, cluster 0 occupies a central position in development trajectory, and is primarily composed of inhibitory neurons, astrocytes, and excitatory neurons. Serving as intermediate state cells, it differentiates into multiple clusters: towards cluster 1 and cluster 6, forming more mature inhibitory and excitatory neurons, as well as astrocytes; towards cluster 2, generating specific functional subgroups of excitatory neurons; towards cluster 3, producing a group that includes ependymal cells, excitatory neurons, inhibitory neurons, and astrocytes, highlighting its role in the interaction between neural development and support cells; towards cluster 4, forming mature oligodendrocyte cells, which participate in myelination; and towards cluster 5, producing a support cell group composed of astrocytes, endothelial cells, and pericytes, underscoring their key roles in regulating the neuronal environment and vascular function [46–48]. These developmental paths reveal the complex differentiation mechanisms and interactions of different types of neurons and support cells in the hypothalamic preoptic region.

### CCC results on the seqFISH+ data of mouse secondary somatosensory cortex

**Fig 4:**
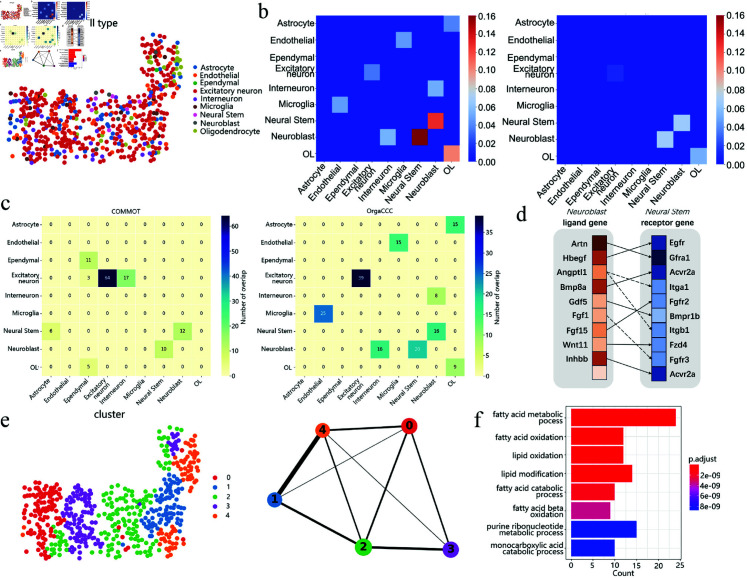
Analysis of cell-cell communication results on seqFISH+ data of mouse secondary somatosensory cortex. **a**. Spatial distribution of the cell types. **b**. Comparison of the cell-type-level cellular communications inferred by OrgaCCC (left) and those directly obtained from the original cell spatial graph (right). Colors represent the communication strength. **c**. Overlap of ligand-receptor pairs predicted by COMMOT (left) with CellChat, iTALK, and OrgaCCC, as well as overlap predicted by OrgaCCC (right) with CellChat, iTALK, and COMMOT. **d**. Predicted ligand-receptor gene pairs mediating communication between neuroblasts and neural stem cells using OrgaCCC. The color from darkest to lightest represents rankings from highest to lowest. Solid lines indicate ligand-receptor pairs also identified by other compared methods, while dashed lines represent pairs uniquely identified by OrgaCCC. **e**. The cell clusters obtained by spectral clustering on the reconstructed cell-level graph ${\hat{A}}_{c}$, and the corresponding UMAP and PAGA graphs. **f**. Bioprocess enrichment analysis of the top-ranked sensitive genes.

The dataset of the mouse secondary somatosensory cortex generated using seqFISH+ consists of 523 cells and 10,000 genes. Fig 4a shows the spatial distribution of the cell types. Fig 4b compares the cell-cell communications predicted using OrgaCCC with those directly derived from the original spatial graph *A*_*c*_. In the original spatial graph *A*_*c*_, only the interactions between neural stem cells and neuroblast cells are preserved, while more interactions were predicted by OrgaCCC, and the signals appear much stronger and more pronounced. The communications identified by OrgaCCC can be broadly grouped into several key groups. Endothelial cells and microglia exhibit significant interactions, reflecting their critical roles within the neurovascular unit. These interactions are essential for the formation, maintenance, and regulation of the blood-brain barrier (BBB) and cerebral blood flow, relying on the functional interplay between endothelial cells and microglia [49]. The communication between neuroblasts, neural stem cells, and interneurons highlights the dynamic process of neuroblast migration and differentiation. Neuroblasts, as undifferentiated precursors, form synaptic connections with interneurons during development. These interneurons may influence neuroblast differentiation by releasing neurotransmitters, thereby modulating the type and proportion of neurons in the cortex [50]. The interaction between astrocytes and OL underscores the importance of chemical signaling in maintaining the integrity and function of the nervous system. These glial cell interactions are essential for regulating cellular morphology, function, and communication between glial cells and neurons [51].

The predicted ligand-receptor pairs mediating communication between each pair of different cell types using OrgaCCC, CellChat, COMMOT, and iTALK were compared. Note that NiCo was not included in the comparison due to its instability when the number of cells is small. Fig 4c illustrates the overlap of predicted ligand-receptor pairs, showing the results of COMMOT and OrgaCCC when compared with those of other methods. Results for CellChat and iTALK are provided in the supplementary materials (S3 Fig b). This comparison evaluates the consistency and uniqueness of predicted interactions, highlighting key mediators of intercellular communication. OrgaCCC demonstrates higher overlap with other methods while also identifying unique ligand-receptor pairs, underscoring its robustness and ability to uncover novel interactions critical for cell-cell communication networks. Fig 4d presents the top ten ligand-receptor pairs predicted by OrgaCCC to mediate communication between neuroblasts and neural stem cells. Notably, OrgaCCC uniquely predicts the ligand-receptor pairs *Angptl1-Itga1*, *Angptl1-Itgb1*, and *Fgf1-Fgfr3*, which suggest important signaling pathways in the communication from neuroblasts to neural stem cells. *Angptl1*, by binding to integrin receptors *Itga1* and *Itgb1*, may regulate neuroblast-extracellular matrix interactions, promoting cell migration, adhesion, and their transformation into neural stem cells [52, 53]. *Fgf1*, by binding to *Fgfr3*, could activate downstream signaling pathways that support neural stem cells proliferation, self-renewal, and neuroblast fate decisions [54]. Together, these signaling pathways may synergistically promote neural development, maintain the balance of the neural stem cell pool, and regulate the transformation of neuroblasts into neural stem cells.

We again applied spectral clustering to the final cell-level graph ${\hat{A}}_{c}$, partitioning it into five clusters. To visualize the potential connections and structures among these cell clusters, we constructed a PAGA plot (Fig 4e). In the mouse secondary somatosensory cortex, the developmental trajectory originates from cluster 2, a multipotent state primarily composed of excitatory neurons, astrocytes, and endothelial cells (S3 Fig c). This state bifurcates into two major pathways: the neuronal differentiation pathway and the vasculature-associated cell differentiation pathway. In the neuronal pathway, cluster 2 first differentiates into cluster 1, which is enriched with excitatory neurons and interneurons, and further differentiates into cluster 4, a mixed state containing more mature neurons and supporting cells. In the vasculature-associated pathway, cluster 2 differentiates into cluster 3, which contains endothelial cells and a small number of excitatory neurons, and then progresses to cluster 0, forming mature vasculature-associated cell populations such as oligodendrocytes (OL) and endothelial cells. These inferred trajectories suggest that cluster 2 serves as the central developmental hub, with its multipotency supporting the formation of multiple differentiation pathways [55, 56].

We further employed sensitivity analysis (see Methods) to select the genes that play crucial roles in cell-cell communications. According to the sensitivity level, we selected the top 10% of genes and performed bioprocess enrichment analysis (Fig 4f). The analysis revealed that these genes are mainly involved in biological processes such as fatty acid metabolic process, fatty acid oxidation and lipid oxidation. Metabolic products, including fatty acid oxidation products, lactate, and ATP, not only provide energy but also function as ligands, interacting with cell surface receptors to regulate intercellular signaling. For example, fatty acid metabolites can modulate cellular functions through G protein-coupled receptors (GPCRs), while purine metabolites like ATP participate in intercellular signaling via P2 receptors, influencing immune responses, neurotransmission, and other processes. Furthermore, changes in metabolic states can regulate the secretion of cytokines, further impacting cell-to-cell communication, especially among immune cells and neurons. These metabolic processes may significantly influence neuronal activity and neuroimmune interactions by modulating receptor expression and cytokine release [57, 58].

### CCC results on the Visium data of mouse cortex

**Fig 5:**
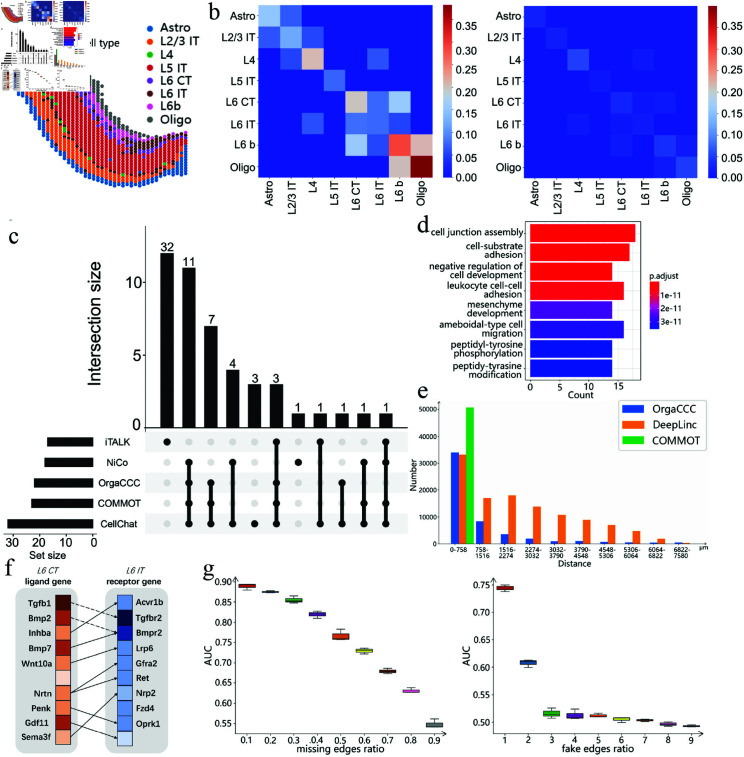
Analysis of cell-cell communication results on Visium data of mouse cortex. **a**. Spatial distribution of the cell types. **b**. Comparison of cell-type-level cellular communications inferred by OrgaCCC (left) and those directly obtained from the original spot spatial graph (right). Colors represent the communication strength. **c**. Comparison of cell-type-level CCCs inferred by OrgaCCC, CellChat, COMMOT, NiCo, iTALK. Numbers count the cell-type-level communication pairs. **d**. Enrichment analysis of the top-ranked significant genes. **e**. Number of the communicating cell pairs at different distances predicted by OrgaCCC, DeepLinc and COMMOT. **f**. Predicted ligand-receptor gene pairs mediating communication between L6 CT and L6 IT neurons using OrgaCCC. The color from darkest to lightest represents rankings from highest to lowest. Solid lines indicate ligand-receptor pairs also identified by other methods (CellChat, COMMOT, NiCo or iTALK), while dashed lines represent pairs uniquely identified by OrgaCCC. **g**. Robustness analysis of OrgaCCC when different ratios of edges in the cell spatial graph *A*_*c*_ were removed and perturbed.

The mouse cortex Visium dataset consists of 1,073 spots and 648 genes. Cell type annotations for each spot were derived from deconvolution results provided in the dataset metadata, where each spot was assigned the cell type with the highest predicted proportion. The spatial distribution of these annotated cell types is shown in Fig 5a. Fig 5b compares the predictions using OrgaCCC with those derived directly from the original spatial graph *A*_*c*_. The results from OrgaCCC reveal more distinct and biologically meaningful interactions between specific cell types. One primary interaction module involves astrocytes (Astro) and neurons. Astrocytes (Astro) and layer 2/3 intratelencephalic neurons (L2/3 IT) exhibit close interactions. Astrocytes can secrete neurotrophic factors such as BDNF and NGF to promote the growth and functionality of L2/3 IT neurons, while L2/3 IT neurons can, in turn, influence the activity and function of astrocytes [59]. Significant communication also exists among layer 6 intratelencephalic neurons (L6 IT), layer 6 corticothalamic neurons (L6 CT), and layer 6b neurons (L6b). L6 IT neurons interact with L6 CT neurons primarily in the areas of information transfer and regulation. L6 IT neurons typically transmit information vertically upward, influencing inputs from lower-level neurons, while L6 CT neurons connect with neurons in other cortical regions to integrate information across brain regions [60]. Furthermore, L6b neurons play a regulatory role in sensory cortical inputs and interregional information transfer and can form connections with both other L6 neurons and neurons in different cortical layers. And L6b neurons interact with oligodendrocytes (Oligo) through synaptic connections. Oligo can release neurotransmitters or signaling molecules to modulate the activity of L6b neurons [61, 62]. The interactions among different cell types obtained from the original spatial graph *A*_*c*_ are relatively sparse, with generally weak connection strengths, failing to clearly reveal the complex communication relationships between cells. In contrast, OrgaCCC is effective at uncovering the true intercellular interactions and regulatory mechanisms with the generative modeling.

We further compared the predicted cell-cell communications with those from CellChat, COMMOT, NiCo, and iTALK, focusing on the overlap of cell-type-level communication pairs (Fig 5c). OrgaCCC identified 22 communication pairs, all of which overlapped with predictions from other methods, demonstrating its high reliability. In comparison, alternative tools exhibited varying degrees of overlap: CellChat predicted 32 pairs (66% overlap), COMMOT detected 23 pairs (100% overlap), NiCo revealed 18 pairs (94% overlap), and iTALK predicted 17 pairs (28% overlap). While the total number of predicted pairs varied across different methods, the substantial overlap highlights the consistency among methods and reinforces the robustness of the OrgaCCC in accurately predicting intercellular communication.

We also analyzed the ligand-receptor pairs mediating communication between different cell types. The results of the overlapping ligand-receptor pairs obtained using OrgaCCC, COMMOT, CellChat, NiCo and iTALK are shown in the supplementary materials (S4 Fig a). We examined the overlap of the ligand-receptor gene pairs predicted by one particular method and the union set predicted by the other four methods. Compared to CellChat, NiCo, COMMOT, and iTALK, OrgaCCC not only shows a higher degree of overlapping, but also predicts some unique ligand-receptor interactions, especially between cell type pairs such as L5 IT and L6 IT. Fig 5f shows the top 10 genes predicted by OrgaCCC involved in communication between L6 CT and L6 IT. Notably, ligand-receptor pairs such as *Tgfb1-Tgfbr2* and *Bmp2-Bmpr2* play critical roles in neurodevelopment and signaling. *Tgfb1*, through its binding to *Tgfbr2*, regulates neuronal development, synaptic plasticity, and axon guidance [63]. *Bmp2*, by interacting with *Bmpr2*, activates downstream pathways that influence neuronal differentiation and axon formation, contributing to the organization of cortical neurons [64]. These signaling pathways work in concert to precisely orchestrate the development, functionality, and maturation of L6 CT and L6 IT neurons, thereby facilitating the establishment of cortical neural circuits.

The top 10% of genes playing most significant roles in the cellular communication process were also selected using sensitivity analysis (see Methods) for enrichment analysis (Fig 5d). This analysis reveals several key biological processes in the mouse cortex. Enriched pathways such as cell junction assembly, cell-substrate adhesion, and cell-cell adhesion highlight the pivotal role of cellular interactions and migration in cortical development and function. The pathway for the negative regulation of cell development points to a tightly regulated control of cell differentiation and proliferation, ensuring proper developmental progression [65, 66]. The ameboid-type cell migration pathway is particularly relevant to cell positioning and movement during neurodevelopment. Overall, these enriched pathways underscore the complexity and significance of cellular interactions, migration, and developmental regulation in cortical cell communication.

The hypothesis that spots in closer proximity are more likely to engage in intercellular communications was also supported by the mouse cortex data (Fig 5e). The number of predicted communicating spot pairs increased as the interspot distance decreased. This indicates that OrgaCCC is not only effective in identifying spatially dependent communications but also capable of explaining interactions across longer distances.

To evaluate the robustness of OrgaCCC, we simulated scenarios where the edges in the spot-level graph were either missing or perturbed by false edges (see Methods). OrgaCCC was initially trained using *A*_*c*_ with missing edges to predict CCC, and its predictions were compared with the CCC network inferred from the complete *A*_*c*_. This process was repeated 30 times, and the resulting box-and-line diagrams for AUCs are shown in Fig 5g. OrgaCCC successfully reconstructed the missing edges, achieving high AUC scores even with up to 40% of edges missing, confirming its robustness. OrgaCCC was then evaluated using the perturbed *A*_*c*_ with false edges (Fig 5g). Notably, even when the number of false edges was twice the number of spots, OrgaCCC produced reliable results. These findings demonstrate that OrgaCCC maintains strong tolerance to random errors and exhibits excellent denoising capabilities in cellular communication relationships.

### CCC results on the Visium data of human intestine

**Fig 6:**
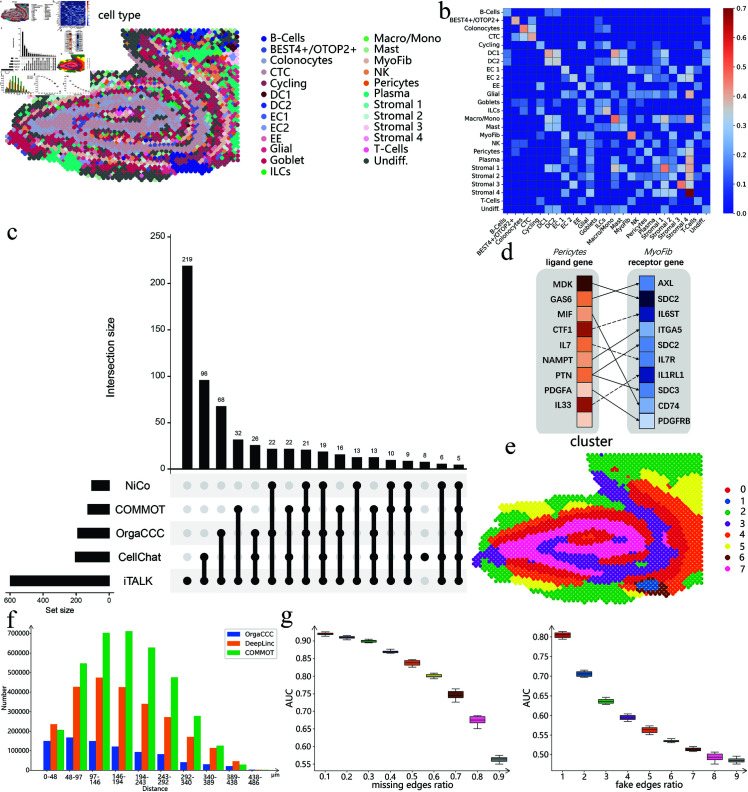
Analysis of cell-cell communication results on Visium data of human intestine. **a**. Spatial distribution of the cell types. **b**. The cell-type-level cellular communications inferred by OrgaCCC. Colors represent the communication strength. **c**. Comparison of cell-type-level CCCs inferred by OrgaCCC, NiCo, CellChat, COMMOT, iTALK. Numbers count the cell-type-level communication pairs. **d**. Predicted ligand-receptor gene pairs mediating communication between pericytes and myofibroblasts cells using OrgaCCC. The color from darkest to lightest represents rankings from highest to lowest. Solid lines indicate ligand-receptor pairs also identified by other methods (NiCo, CellChat, COMMOT, or iTALK), while dashed lines represent pairs uniquely identified by OrgaCCC. **e**. The cell clusters obtained by spectral clustering on the reconstructed spot-level graph ${\hat{A}}_{c}$. **f**. Number of the communicating cell pairs at different distances predicted by OrgaCCC, DeepLinc and COMMOT. **g**. Robustness analysis of OrgaCCC when different proportions of edges in the cell spatial graph *A*_*c*_ were removed and perturbed.

The human intestine Visium dataset consists of 2,649 spots and 15,882 genes, with the spatial distribution of the cell types shown in Fig 6a. Cell type annotations for each spot were derived from deconvolution results provided in the dataset metadata, where each spot was assigned the cell type with the highest predicted proportion. Fig 6b illustrates the cell-type-level communications identified using OrgaCCC. In the human fetal intestine, cellular communication can be divided into five functional groups: immune cell populations including B cells, T cells, dendritic cells (DC), and macrophages (Macro) establish immune surveillance, tolerance, and coordination between innate and adaptive immunity, laying the foundation for postnatal defense [67]. Epithelial cell populations such as colonocytes, goblet cells, and enteroendocrine (EE) cells maintain microenvironmental stability through barrier formation, mucus secretion, and hormonal regulation [68]. Stromal-vascular cell populations including endothelial cells (EC), pericytes, and myofibroblasts (MyoFib) support tissue architecture development through angiogenesis and ECM remodeling while regulating the stem cell niche [69]. Neuronal-glial cell populations like glial cells and certain stromal cells collaborate with the enteric nervous system to promote peristaltic reflexes and neuro-immune crosstalk [70]. Proliferation-associated cell populations including cycling cells and undifferentiated (Undiff.) cells drive rapid tissue expansion and differentiation reserves [71]. These groups coordinate through immune-epithelial crosstalk, stromal-epithelial signaling, and vascular-neural interactions, collectively ensuring the fetal intestine’s barrier function, immune tolerance, vascularization, and neural innervation to secure postnatal digestive and immune homeostas.

The cell-type-level CCCs predicted by OrgaCCC, CellChat, COMMOT, iTALK and NiCo were then compared. Fig 6c summarizes the comparative results. OrgaCCC identified 190 cell-type-level communication pairs, showing significant overlap with predictions from established methods. Specifically, CellChat predicted 204 pairs (96% overlap), while iTALK identified 597 pairs (63% overlap). This strong concordance validates OrgaCCC’s reliability for intercellular communication analysis.

The evaluation of the overlapping ligand-receptor pairs predicted by the compared methods (CellChat, COMMOT, NiCo and iTALK) revealed that OrgaCCC achieved superior overlap rates while uniquely identifying interactions in key cell type pairs like pericytes and myoFib cells (S5 Fig a). Fig 6d shows the top 10 genes predicted by OrgaCCC involved in communication between pericytes and myoFib cells. For example, the *IL33*-*IL1RL1* pair likely serves as an important immune-stromal interface, where the cytokine *IL33* , acting as an “alarmin” released upon cellular stress or tissue damage, signals through the ST2 receptor (*IL1RL1*) to modulate type 2 immune responses and fibroblast activation [72].

We performed spectral clustering on the final spot-level graph ${\hat{A}}_{c}$, dividing it into eight clusters (Fig 6e). We then performed UMAP for low-dimensional visualization and applied PAGA to elucidate topological relationships between distinct cell populations (S5 Fig b). These newly defined cell clusters revealed intercellular communication relationships and tissue developmental progression. S5 Fig d displays the dominant cell types comprising each cluster. For example, cluster 0 primarily contains cycling cells, goblet cells and undifferentiated cells, which differentiate along two distinct lineages. In the epithelial lineage, they first transition through cluster 2 (characterized by undifferentiated cells, goblet cells, and plasma cells), then progressively mature into cluster 3 enriched by markers including crypt top colonocytes and BEST4+/OTOP2+ cells, before ultimately differentiating into terminal colonocytes (cluster 7) [73]. Concurrently, in the mesenchymal-immune lineage, these progenitor cells develop through cluster 4 (enriched with myoFib and glial cells), further differentiating into cluster 5 (containing plasma cells and stromal 3 cells), thereby contributing to key functional units of the intestinal microenvironment [26, 74]. This differentiation trajectory fully reveals the dynamic process of intestinal cell development from pluripotent states to terminally differentiated functional cells.

Analysis of the human intestine data (Fig 6f) further validated the spatial dependency of cell-cell interactions, showing that communication events occurred more frequently between nearby spots and gradually decreased as the distance between spots increased. OrgaCCC successfully captured both short-range signaling between neighboring spots and biologically relevant long-distance interactions, demonstrating its comprehensive detection capability for spatially organized cellular networks.

To assess the stability of OrgaCCC, we conducted systematic perturbation analyses by either randomly removing edges from or introducing false edges into the spot-level graph (see Methods). First, we conducted 30 independent trials to randomly remove different proportions of edges from the adjacency matrix *A*_*c*_ and evaluated the ability of OrgaCCC to recover the original CCC network. Fig 6g demonstrates the excellent edge reconstruction capability of OrgaCCC. We further tested OrgaCCC on perturbed graphs containing false edges. When the number of false edges is proportionally low, OrgaCCC can still make reliable predictions. These results highlight OrgaCCC’s robust performance against random noise interference and its superior denoising capacity.

## Discussion

In this study, we introduce OrgaCCC, a novel framework designed to infer cell-cell communication networks by leveraging two orthogonal graph autoencoders: one operating at the cell/spot level and the other at the gene level. By integrating spatial transcriptomics data with ligand-receptor interactions, OrgaCCC effectively captures both local cell microenvironments and the dynamics of cell-cell communication, resulting in a comprehensive and biologically relevant map of cellular interactions. When OrgaCCC was applied to multiple spatial transcriptomics datasets, and compared with state-of-the-art methods, it demonstrates its superiority in successfully inferring communication at the cell-type, cell, and ligand-receptor levels. The predictions at both the cell-type and ligand-receptor levels exhibit high consistency with those inferred by other methods, while uniquely identifying additional interactions of biological significance. Spectral clustering of the resulting cell/spot-level communication networks reveals clear patterns of communications and developmental trajectories among different cell types. Furthermore, the significant genes identified through sensitivity analysis are shown to be involved in key biological processes. Robustness analysis confirms that OrgaCCC maintains high prediction accuracy even when dealing with noisy or incomplete data, highlighting its reliability and applicability to diverse datasets. OrgaCCC offers valuable insights into cellular interactions, advancing our understanding of tissue organization and function in various biological contexts.

Although OrgaCCC has made significant progress in CCC inference, there are still areas that are worth further exploration and improvement. First, OrgaCCC currently focuses primarily on ligand-receptor gene interactions, but cell communication encompasses a broader range of interactions. Future research could enhance the model by incorporating data on transcription factors, signaling molecules, and other factors to construct a more comprehensive and multilayered gene network. This would improve prediction accuracy, enhance model interpretability, and provide deeper insights into the interplay between intracellular regulatory networks and cell-cell communications. Additionally, OrgaCCC mainly focuses on static communication networks, but cell communication is inherently dynamic, particularly during development, immune responses, and disease progression. Future studies could incorporate temporal dimensions to construct dynamic cell communication networks, capturing communication changes across different physiological states and developmental stages. Finally, the ligand-receptor gene networks ${\hat{A}}_{g}$ reconstructed by OrgaCCC have not yet been fully exploited. Future research could explore their potential in revealing gene interactions and regulatory mechanisms involved in cell-cell communications.

## Materials and methods

### Cell/Spot-level and gene-level graph construction

Given the gene expression matrix $X\in {\mathbb{R}}^{m\times n}$ corresponding to *m* genes and *n* cells/spots in the spatial transcriptomics data, and the spatial locations, the cell/spot-level graph is constructed by calculating the pairwise spatial Euclidean distance between cells/spots. The adjacency matrix *A*_*c*_ is defined as *A*_*c*_(*i*,*j*) = 1 if the distance between cell/spot *i* and cell/spot *j* is smaller than a predefined distance threshold, indicating that the two cells/spots are considered neighbors; otherwise, *A*_*c*_(*i*,*j*) = 0. This graph is denoted as ${𝒢}_{c}=(A_{c},X^{T})$. The distance threshold was heuristically selected to ensure that the majority of cells have at least three spatial neighbors, consistent with the biological assumption that most cells in a tissue are in contact with several adjacent cells. To ensure consistency across datasets, the same distance-based approach was uniformly applied to both single-cell and spot-level spatial transcriptomics data. The structure of this graph reflects the local microenvironment surrounding each cell/spot, facilitating the reconstruction of cell-specific gene expression while preserving spatial information. From the perspective of cell-cell communication, this graph encodes physical interactions between cells/spots, providing a partial and noisy subset of the full cell-cell communication network.

The gene-level graph ${𝒢}_{g}=(A_{g},X)$ among the *m* genes is directly constructed based on ligand-receptor interactions derived from the CellChat database [3]. $A_{g}\in {\mathbb{R}}^{m\times m}$ denotes the ligand-receptor interactions. The structure of this graph captures the functional relationships between genes as mediated through ligand-receptor interactions, enabling the study of gene expression patterns within the context of cellular communication. Since CellChatDB considers multimeric complexes of ligands and receptors, an interaction between ligand and receptor genes is established in *A*_*g*_ only when both ligand and receptor genes are simultaneously expressed.

### Gene-level and cell/spot-level variational graph autoencoders

Since the gene-level graph ${𝒢}_{g}=(A_{g},X)$ encodes directed ligand-receptor interactions across different cells/spots, we employ variational directed graph autoencoders (VDGAE) [20]. The directionality in ${𝒢}_{g}$ reflects the biological signaling flow from ligand genes to their corresponding receptor genes, and each directed edge in *A*_*g*_ denotes a known ligand-receptor interaction. The initial cell/spot-level graph is undirected as is constructed from the spatial information. Our goal is to reconstruct a directed cell-cell communication graph that integrates both cell/spot spatial relationships and ligand-receptor interactions. Therefore, OrgaCCC jointly learns two orthogonal graph autoencoders, which are entangled to effectively capture both aspects of the data.

Without loss of generality, we consider a directed graph (*A*,*X*), where *A* is the adjacency matrix of *N* nodes, and *X* represents the feature matrix with the number of rows being *N*. In OrgaCCC, *N* equals to *m* for gene-level graph and *n* for cell/spot-level graph. The variational directed graph encoder aims to approximate the distribution p(Z|A,X) in the latent space by a two-layer graph convolutional network (GCN), where $Z=[z_{1},\ldots ,z_{N}{]}^{T}\in {\mathbb{R}}^{N\times d}$ consists of *d*-dimensional latent vector $z_{i}\in {\mathbb{R}}^{d}$ for each node *i*. The true distribution for p(Z|A,X) is typically complex and intractable, thus variational inference is used to approximate it, which is modeled by q(Z|A,X) as


$$q(Z|A,X)=\prod_{i=1}^{N}q(z_{i}|A,X),$$

with $q(z_{i}|A,X)=𝒩(z_{i}|\mu_{i},diag(\sigma_{i}^{2}))$, where the mean vectors $\mu_{i}$ form a mean matrix $\mu ={GCN}_{\mu }(A,X)$, and the standard deviation matrix corresponds to $\log\sigma ={GCN}_{\sigma }(A,X)$. The two-layer GCN for a directed graph (*A*,*X*) is defined as $GCN(A,X)=\tilde{A}\text{ReLU}(\tilde{A}XW_{0})W_{1}$, where *W*_*i*_ denotes the weight matrices, and ReLU(·)=max(0,·). The matrix $\tilde{A}$ is the Laplacian matrix defined as $D_{\text{out}}^{-1}(A\;+\;I)$ for a directed graph, with $D_{\text{out}}$ being the diagonal out-degree matrix of *A* + *I*. Note that in the two-layer GCNs, ${GCN}_{\mu }(A,X)$ and ${GCN}_{\sigma }(A,X)$ share the first-layer parameter *W*_0_.

The graph decoder reconstructs the directed graph based on the latent vectors *z*_*i*_ sampled from the posterior distribution, formulated as:

$$p(\hat{A}|Z)=\prod_{i=1}^{N}\prod_{j=1}^{N}p({\hat{A}}_{ij}|z_{i},z_{j}).$$ (1)

We employ a directed graph decoder as proposed in the work [20], given by $p({\hat{A}}_{ij}=1|z_{i},z_{j})=\sigma (z_{i}^{(s)T}z_{j}^{\left(t\right)}),$ where $z_{i}^{\left(s\right)}=z_{i\left[1:\frac{d}{2}\right]}$ and $z_{j}^{\left(t\right)}=z_{j\left[(\frac{d}{2}+1):d\right]}$. This formulation preserves higher-order node proximity and captures asymmetric transmissibility. Specifically, for an even dimension *d*, the first *d*/2 dimensions represent the source node vectors, while the remaining *d*/2 dimensions correspond to the target node vectors.

Besides the graph reconstruction using graph decoder, the gene expression matrix is also reconstructed from the same latent vectors *z*_*i*_, utilizing a two-layer fully-connected network. The reconstructed cell-cell communication matrix ${\hat{A}}_{c}$ represents the strength of the communication between the cells/spots.

### Loss function for OrgaCCC

OrgaCCC jointly learns the orthogonal graph autoencoders through a loss function composed of three components: a gene-level loss $L_{\text{gene}}$, a cell/spot-level loss *L*_*cell*_, and a coupled orthogonal loss *L*_*orth*_.

The gene-level loss $L_{\text{gene}}$ consists of the gene-level graph reconstruction loss $L_{A_{g}}$ and the gene expression reconstruction loss $L_{X_{g}}$. Specifically, the graph is reconstructed by maximizing a tractable variational lower bound (ELBO)

$$L_{A_{g}}={𝔼}_{q(Z_{g}|A_{g},X)}\left[\log p(A_{g}|Z_{g})\right]-{𝒟}_{KL}(q(Z_{g}|A_{g},X)||p(Z_{g})),$$ (2)

with a Gaussian prior $p(Z_{g})=\prod_{i}p(z_{gi})=\prod_{i}𝒩(z_{gi}|0,1)$, where ${𝒟}_{KL}$ is the Kullback-Leibler divergence. The gene expression matrix ${\hat{X}}_{g}$ is reconstructed by minimizing the following loss function:

$$L_{X_{g}}=\frac{1}{mn}\|X-{\hat{X}}_{g}{\|}_{F}^{2},$$ (3)

where $\|\;\cdot \;{\|}_{F}$ denotes the Frobenius norm, and *X* and ${\hat{X}}_{g}$ represent the original and reconstructed gene expression data, respectively.

Similarly, the cell/spot-level loss $L_{\text{cell}}$ consists of the graph reconstruction loss $L_{A_{c}}$ and the gene expression reconstruction loss $L_{X_{c}}$. The loss function for reconstructing cell/spot-level graph is:

$$L_{A_{c}}={𝔼}_{q(Z_{c}|A_{c},X^{T})}\left[\log p(A_{c}|Z_{c})\right]-{𝒟}_{KL}(q(Z_{c}|A_{c},X^{T})||p(Z_{c})),$$ (4)

where $p(Z_{c})=\prod_{i}p(z_{ci})=\prod_{i}𝒩(z_{ci}|0,1)$. The gene expression matrix ${\hat{X}}_{c}$ is reconstructed by minimizing:

$$L_{X_{c}}=\frac{1}{nm}\|X-{\hat{X}}_{c}{\|}_{F}^{2}.$$ (5)

In order to integrate the information of both the cells/spots and the genes, we assume that the gene expression matrix ${\hat{X}}_{g}\in {\mathbb{R}}^{m\times n}$ reconstructed from the gene-level graph $𝒢g=(A_{g},X)$ should be consistent with the gene expression matrix ${\hat{X}}_{c}\in {\mathbb{R}}^{n\times m}$ reconstructed from the cell/spot-level graph $𝒢c=(A_{c},X^{T})$. Similar to the loss proposed in the work [21], we first compute cell-cells/spot-spot correlation matrix *S* between the cells/spots reconstructed from cell/spot-level and gene-level autoencoders as follows:

$$S_{ij}=\frac{\langle {\hat{X}}_{c}(\cdot ,i),{\hat{X}}_{g}^{T}(j,\cdot )\rangle }{\left||{\hat{X}}_{c}(\cdot ,i)||||{\hat{X}}_{g}^{T}(j,\cdot )|\right|},\forall i,j\in [1,n],$$ (6)

where ⟨·,·⟩ represents inner product of two column vectors. *S*_*ij*_ denotes the cosine similarity between the reconstructed gene expression ${\hat{X}}_{c}(\cdot ,i)$ for the *i*th cell/spot in ${\hat{X}}_{c}$ and the reconstructed gene expression ${\hat{X}}_{g}(\cdot ,j)$ for the *j*th cell/spot in ${\hat{X}}_{g}$. OrgaCCC forces the orthogonal correlation matrix *S* to be close to the identity matrix $I\in {\mathbb{R}}^{n\times n}$, by the orthogonal loss

$$L_{\text{orth}}=\frac{1}{n^{2}}\sum (S-I{)}^{2}\;=\frac{1}{n}\sum_{i=1}^{n}{\left(S_{ii}-1\right)}^{2}+\frac{1}{n^{2}-n}\sum_{i=1}^{n}\sum_{j\neq i}{\left(S_{ij}\right)}^{2}.$$ (7)

The overall loss function of OrgaCCC to learn the orthogonal graph autoencoders is:

$$L=L_{\text{gene}}+L_{\text{cell}}+L_{\text{orth}}.$$ (8)

### Cell-type-level cell-cell communication inference

The reconstructed cell/spot-level graph ${\hat{A}}_{c}$ is generated by integrating gene expression, spatial information of the cells/spots, and ligand-receptor interactions, and is thus considered to represent the communication strength among cells/spots. To elucidate the communication relationships between different cell types, ${\hat{A}}_{c}$ is further aggregated into the cell-type-level communication matrix ${\hat{A}}^{cl}$. For spatial transcriptomics datasets generated by spot-based platforms (e.g., 10x Visium), where each spot may contain a mixture of cell types, we assigned a predominant cell-type label to each spot based on deconvolution results or the majority cell type inferred from reference data. Specifically, the communication strength from cell type *k* to cell type *l* is defined as the average communication weights from all cells belonging to cell type *k* to all cells belonging to cell type *l*. The *p*-value measuring significance of cell-cell communication at the cell-type-level is assessed by performing multiple independent random permutations of cell type labels and calculating the percentile of the observed communication strengths within the distribution of communication strengths generated from the randomized label arrangements.

### Identifying significant genes in cell-cell communications

To identify genes critical for cell-cell communication, we performed sensitivity analysis. For each gene, we perturbed its expression across all cells/spots to create the modified profile, which was then fed into OrgaCCC to generate the CCC network and calculate the average precision (AP) value. Here, the AP value is defined as the area under the precision-recall curve, where intercellular communication edges are ranked by their predicted probabilities. Precision is calculated as the proportion of predicted communication edges that are also present in the original cell/spot graph *A*_*c*_, while recall is the proportion of edges in *A*_*c*_ that are correctly identified by the prediction. These values are computed at different thresholds to construct the precision-recall curve. The higher the AP value, the better the model performs in recovering the edges in the original cell/spot graph. We then compared AP values from perturbed profiles with those from unmodified baseline profiles. The absolute difference between these values is designated as the gene’s sensitivity score, quantifying its influence on model performance: larger differences signify greater contributions to cell-cell communication networks and higher functional importance.

This sensitivity analysis enables a systematic evaluation of the contribution of individual genes to the overall communication network. Genes with high sensitivity values are likely to serve as key drivers of intercellular signaling, acting as critical mediators in processes such as ligand-receptor interactions, signal transduction, or transcriptional regulation. Identifying these genes provides valuable insights into the molecular mechanisms underlying tissue organization, developmental processes, and disease progression.

### Model robustness analysis

In spatial transcriptomics, inaccuracy of cell/spot localizations can introduce biases when constructing the cell/spot-level graph. To evaluate the robustness of OrgaCCC, we adopted a methodology inspired by DeepLinc [18], which involves artificially modifying the cell/spot-level graph under two scenarios: randomly removing and perturbing a proportion of edges. These simulations are designed to assess the model’s denoising capabilities and its performance under varying levels of missing data.

First, we systematically remove a certain percentage of edges from the original cell/spot-level graph and train the model on the remaining subgraph. Subsequently, the cell-cell communication relationships predicted by the model using the modified graph are compared with those predicted using the original graph. The model’s performance is evaluated by calculating the Area Under the ROC Curve (AUC). The AUC metric measures the model’s ability to distinguish between existing communication relationships (positive samples) and non-existing communication relationships (negative samples), thereby assessing the model’s predictive accuracy and robustness under conditions of data loss. Furthermore, to evaluate the robustness of OrgaCCC in inferring intercellular communications, we introduce false edges between randomly selected cell/spot pairs in the original cell/spot spatial graph. The model is then trained on this perturbed network, and the resulting AUC values are computed to evaluate its ability to filter out noise and accurately recover cell-cell communication relationships. Specifically, the AUC compares the predicted graph, obtained from training on the perturbed network, with the original OrgaCCC predictions, thereby quantifying the model’s performance in distinguishing true communication from noise. These experiments are repeated 30 times at different perturbation ratios and the results are visualized using box plots.

### Implementation details and parameter settings

#### OrgaCCC

For cell/spot-level graph construction, it is biologically reasonable to assume that most cells in a two-dimensional tissue are in direct contact with at least three neighboring cells [18]. Based on this assumption, we heuristically selected a minimal distance threshold for each dataset such that over 80% of cells have at least three neighbors within the specified range. Specifically, the distance thresholds were set as follows: 300 μm for the STARmap dataset of mouse placenta; 40 μm for the MERFISH dataset of the mouse hypothalamic preoptic region; 200 μm for both the seqFISH+ dataset of the mouse secondary somatosensory cortex and the Visium dataset of the mouse cortex; and 10 μm for the Visium dataset of the human intestine. All other hyperparameters were kept consistent across datasets and set to the default values used in our code implementation.

#### CellChat

CellChat 2.1.2 was used with the default CellChatDB ligand–receptor interaction database. The Function ‘computeCommunProb’ was applied with the following parameters: type = truncatedMean, trim = 0.1, distance.use = TRUE, interaction.range = 250, scale.distance = 0.01, contact.dependent = TRUE, contact.range = 100, and nboot = 20; ‘filterCommunication’ used min.cells = 10.

#### COMMOT

COMMOT version 0.0.3 was used in the analysis. The ligand–receptor database was generated using the function ‘commot.pp.ligand_receptor_database’ with database = CellChat. Cell–cell communication inference was performed using ‘commot.tl.spatial_communication’ with the parameters database_name = cellchat, dis_thr = 500, heteromeric = True, and pathway_sum = True.

#### NiCo

NiCo version 1.4.0 was used with the default ligand–receptor database. Cell–cell interactions were inferred using the function ‘sint.spatial_neighborhood_analysis’ with the parameter Radius = 0.

#### iTALK

iTALK was used with its default ligand–receptor interaction database. Highly expressed genes were identified using the function ‘rawParse’ with top_genes = 50 and stats = mean. Ligand–receptor pairs were detected using the function ‘FindLR’ with datatype = mean count and communication types set as growth factor, other, cytokine, and checkpoint.

#### DeepLinc

DeepLinc version 1.0.0 was used with default parameter settings.

## Supporting information

S1 FigDownstream analysis on STARmap data of the mouse placenta.**a**, Comparison of cellular communication predicted by OrgaCCC (left) and directly obtained from the original cell spatial graph (right). Colors represent the communication strength, with red indicating stronger interactions and blue indicating weaker interactions. **b**, Simulation of missing edges in cell spatial graph. Randomly remove different proportions of real edges in the cell spatial graph, bring them into the model training to get the AUC value, repeat the process thirty times and plot the boxplots. **c**, Cell types mainly contained in each cluster obtained by spectral clustering of cell graph ${\hat{A}}_{c}$. **d**, The overlap of ligand-receptor pairs predicted by CellChat with COMMOT, iTALK, NiCo and OrgaCCC, by iTALK with CellChat, OrgaCCC, NiCo and COMMOT, and by NiCo with CellChat, OrgaCCC, iTALK and COMMOT.(PDF)

S2 FigDownstream analysis on MERFISH data of mouse hypothalamic preoptic region.**a**, Comparison of cellular communication predicted by OrgaCCC (left) and the original cell spatial graph (right). **b**, Predicting the number of cell pairs with intercellular communication relationships at different distances by OrgaCCC, DeepLinc and COMMOT. **c**, Cell types in each cluster from spectral clustering of cell graph ${\hat{A}}_{c}$. **d**, The overlap of ligand-receptor pairs predicted by CellChat with COMMOT, iTALK, NiCo and OrgaCCC, by iTALK with CellChat, OrgaCCC, NiCo and COMMOT, and by NiCo with CellChat, OrgaCCC, iTALK and COMMOT. **e**, The top eight biological processes obtained by enrichment analysis using the top sensitive partial genes. **f**, Simulation of missing edges and add fake edges in cell spatial graph. Randomly remove different proportions of real edges or add fake edges in the cell spatial graph, bring them into the model training to get the AUC value, repeat the process thirty times and plot the boxplots.(PDF)

S3 FigDownstream analysis on seqFISH+ data of mouse secondary somatosensory cortex.**a**, Celltype-level of OrgaCCC, CellChat, COMMOT, iTALK prediction of the overlap of results. Numbers represent the pairs of predictions that overlap at the celltype-level. **b**, The overlap of ligand-receptor pairs predicted by CellChat (top) with COMMOT, iTALK, and OrgaCCC, and by iTALK (bottom) with CellChat, OrgaCCC, and COMMOT. **c**, Cell types mainly contained in each cluster obtained by spectral clustering of cell graph ${\hat{A}}_{c}$. **d**, Predicting the number of cell pairs with intercellular communication relationships at different distances by OrgaCCC, DeepLinc and COMMOT. **e**, Simulation of missing edges and add fake edges in cell spatial graph. Randomly remove different proportions of real edges or add fake edges in the cell spatial graph, bring them into the model training to get the AUC value, repeat the process thirty times and plot the boxplots.(PDF)

S4 FigDownstream analysis on Visium data of mouse cortex.**a**, The overlap of ligand-receptor pairs predicted by CellChat with NiCo, COMMOT, iTALK, and OrgaCCC, by iTALK with CellChat, NiCo, OrgaCCC, and COMMOT, by NiCo with CellChat, COMMOT, OrgaCCC, and iTALK, by COMMOT with CellChat, NiCo, OrgaCCC, and iTALK, and by OrgaCCC with CellChat, COMMOT, NiCo, and iTALK, respectively. **b**, The cell clustering results obtained by spectral clustering of cell graph ${\hat{A}}_{c}$, and the corresponding UMAP and PAGA graphs. **c**, Cell types mainly contained in each cluster obtained by spectral clustering of cell graph ${\hat{A}}_{c}$.(PDF)

S5 FigDownstream analysis on Visium data of human intestine.**a**, The overlap of ligand-receptor pairs predicted by CellChat with NiCo, COMMOT, iTALK, and OrgaCCC, by iTALK with CellChat, NiCo, OrgaCCC, and COMMOT, by NiCo with CellChat, COMMOT, OrgaCCC, and iTALK, by COMMOT with CellChat, NiCo, OrgaCCC, and iTALK, and by OrgaCCC with CellChat, COMMOT, NiCo, and iTALK, respectively. **b**, The Cell clustering related UMAP and PAGA graphs. **c**, The top eight biological processes obtained by enrichment analysis using the top sensitive partial genes. **d**, Cell types mainly contained in each cluster obtained by spectral clustering of cell graph ${\hat{A}}_{c}$.(PDF)

## References

[1] Armingol E, Officer A, Harismendy O, Lewis NE. Deciphering cell-cell interactions and communication from gene expression. Nat Rev Genet. 2021;22(2):71–88. doi: 10.1038/s41576-020-00292-x 33168968 PMC7649713

[2] Efremova M, Vento-Tormo M, Teichmann SA, Vento-Tormo R. CellPhoneDB: inferring cell-cell communication from combined expression of multi-subunit ligand-receptor complexes. Nat Protoc. 2020;15(4):1484–506. doi: 10.1038/s41596-020-0292-x 32103204

[3] Jin S, Guerrero-Juarez CF, Zhang L, Chang I, Ramos R, Kuan C-H, et al. Inference and analysis of cell-cell communication using CellChat. Nat Commun. 2021;12(1):1088. doi: 10.1038/s41467-021-21246-9 33597522 PMC7889871

[4] Türei D, Valdeolivas A, Gul L, Palacio-Escat N, Klein M, Ivanova O, et al. Integrated intra- and intercellular signaling knowledge for multicellular omics analysis. Mol Syst Biol. 2021;17(3):e9923. doi: 10.15252/msb.20209923 33749993 PMC7983032

[5] Wang Y, Wang R, Zhang S, Song S, Jiang C, Han G, et al. iTALK: an R package to characterize and illustrate intercellular communication. Cold Spring Harbor Laboratory. 2019. 10.1101/507871

[6] Browaeys R, Saelens W, Saeys Y. NicheNet: modeling intercellular communication by linking ligands to target genes. Nat Methods. 2020;17(2):159–62. doi: 10.1038/s41592-019-0667-5 31819264

[7] Wang X, Almet AX, Nie Q. Detecting global and local hierarchical structures in cell-cell communication using crosschat. Nat Commun. 2024;15(1):1–16. 39627184 10.1038/s41467-024-54821-x PMC11615294

[8] Shen X, Zhao Y, Wang Z, Shi Q. Recent advances in high-throughput single-cell transcriptomics and spatial transcriptomics. Lab Chip. 2022;22(24):4774–91. doi: 10.1039/d2lc00633b 36254761

[9] Tian L, Chen F, Macosko EZ. The expanding vistas of spatial transcriptomics. Nat Biotechnol. 2023;41(6):773–82. doi: 10.1038/s41587-022-01448-2 36192637 PMC10091579

[10] Armingol E, Baghdassarian HM, Martino C, Perez-Lopez A, Aamodt C, Knight R. Context-aware deconvolution of cell-cell communication with tensor-cell2cell. Nat Commun. 2022;13(1):3665. 35760817 10.1038/s41467-022-31369-2 PMC9237099

[11] Garcia-Alonso L, Handfield L-F, Roberts K, Nikolakopoulou K, Fernando RC, Gardner L, et al. Mapping the temporal and spatial dynamics of the human endometrium in vivo and in vitro. Nat Genet. 2021;53(12):1698–711. doi: 10.1038/s41588-021-00972-2 34857954 PMC8648563

[12] Troul´e K, Petryszak R, Cakir B, Cranley J, Harasty A, Prete M, et al. Cellphonedb v5: inferring cell–cell communication from single-cell multiomics data. Nat Protocols. 2025:1–29. 40133495 10.1038/s41596-024-01137-1

[13] Jin S, Plikus MV, Nie Q. CellChat for systematic analysis of cell-cell communication from single-cell transcriptomics. Nat Protoc. 2025;20(1):180–219. doi: 10.1038/s41596-024-01045-4 39289562

[14] Agrawal A, Thomann S, Basu S, Grün D. NiCo identifies extrinsic drivers of cell state modulation by niche covariation analysis. Nat Commun. 2024;15(1):10628. doi: 10.1038/s41467-024-54973-w 39639035 PMC11621405

[15] Dries R, Zhu Q, Dong R, Eng C-HL, Li H, Liu K, et al. Giotto: a toolbox for integrative analysis and visualization of spatial expression data. Genome Biol. 2021;22(1):78. doi: 10.1186/s13059-021-02286-2 33685491 PMC7938609

[16] Cang Z, Zhao Y, Almet AA, Stabell A, Ramos R, Plikus MV, et al. Screening cell-cell communication in spatial transcriptomics via collective optimal transport. Nat Methods. 2023;20(2):218–28. doi: 10.1038/s41592-022-01728-4 36690742 PMC9911355

[17] Li Z, Wang T, Liu P, Huang Y. Spatialdm for rapid identification of spatially co-expressed ligand–receptor and revealing cell–cell communication patterns. Nat Commun. 2023;14(1):3995. 37414760 10.1038/s41467-023-39608-w PMC10325966

[18] Li R, Yang X. De novo reconstruction of cell interaction landscapes from single-cell spatial transcriptome data with DeepLinc. Genome Biol. 2022;23(1):124. doi: 10.1186/s13059-022-02692-0 35659722 PMC9164488

[19] Zhu J, Wang Y, Chang WY, Malewska A, Napolitano F, Gahan JC, et al. Mapping cellular interactions from spatially resolved transcriptomics data. Nat Methods. 2024;21(10):1830–42. doi: 10.1038/s41592-024-02408-1 39227721

[20] Salha G, Limnios S, Hennequin R, Tran V-A, Vazirgiannis M. Gravity-inspired graph autoencoders for directed link prediction. Proc ACM Int Conf Inf Knowl Manag. 2019;589–98.

[21] Liu Y, Tu W, Zhou S, Liu X, Song L, Yang X, et al. Deep graph clustering via dual correlation reduction. AAAI. 2022;36(7):7603–11. doi: 10.1609/aaai.v36i7.20726

[22] He Y, Tang X, Huang J, Ren J, Zhou H, Chen K, et al. ClusterMap for multi-scale clustering analysis of spatial gene expression. Nat Commun. 2021;12(1):5909. doi: 10.1038/s41467-021-26044-x 34625546 PMC8501103

[23] Moffitt JR, Bambah-Mukku D, Eichhorn SW, Vaughn E, Shekhar K, Perez JD. Molecular, spatial, and functional single-cell profiling of the hypothalamic preoptic region. Science. 2018;362(6416):eaau5324. 10.1126/science.aau5324 PMC6482113 30385464

[24] Eng C-HL, Lawson M, Zhu Q, Dries R, Koulena N, Takei Y, et al. Transcriptome-scale super-resolved imaging in tissues by RNA seqFISH. Nature. 2019;568(7751):235–9. doi: 10.1038/s41586-019-1049-y 30911168 PMC6544023

[25] Xu Z, Wang W, Yang T, Li L, Ma X, Chen J, et al. Stomicsdb: a comprehensive database for spatial transcriptomics data sharing, analysis and visualization. Nucleic Acids Res. 2024;52(D1):D1053-D1061. 10.1093/nar/gkad933 PMC10767841 37953328

[26] Fawkner-Corbett D, Antanaviciute A, Parikh K, Jagielowicz M, Gerós AS, Gupta T, et al. Spatiotemporal analysis of human intestinal development at single-cell resolution. Cell. 2021;184(3):810-826.e23. doi: 10.1016/j.cell.2020.12.016 33406409 PMC7864098

[27] Cartwright JE, Balarajah G. Trophoblast interactions with endothelial cells are increased by interleukin-1beta and tumour necrosis factor alpha and involve vascular cell adhesion molecule-1 and alpha4beta1. Exp Cell Res. 2005;304(1):328–36. doi: 10.1016/j.yexcr.2004.11.013 15707597

[28] Curtis MB, Kelly N, Hughes CCW, George SC. Organotypic stromal cells impact endothelial cell transcriptome in 3D microvessel networks. Sci Rep. 2022;12(1):20434. doi: 10.1038/s41598-022-24013-y 36443378 PMC9705391

[29] Xu L, Li Y, Sang Y, Li D-J, Du M. Crosstalk between trophoblasts and decidual immune cells: the cornerstone of maternal-fetal immunotolerance. Front Immunol. 2021;12:642392. doi: 10.3389/fimmu.2021.642392 33717198 PMC7947923

[30] Thomas JR, Naidu P, Appios A, McGovern N. The ontogeny and function of placental macrophages. Front Immunol. 2021;12:771054. doi: 10.3389/fimmu.2021.771054 34745147 PMC8566952

[31] Zhao X, Tian G, Badillo A, Ju W, Zhong N. Single-cell transcriptomics of pathological pregnancies. Placenta Reprod Med. 2023;2.

[32] Cross JC. How to make a placenta: mechanisms of trophoblast cell differentiation in mice--a review. Placenta. 2005;26 Suppl A:S3-9. doi: 10.1016/j.placenta.2005.01.015 15837063

[33] Jiang X, Wang Y, Xiao Z, Yan L, Guo S, Wang Y, et al. A differentiation roadmap of murine placentation at single-cell resolution. Cell Discov. 2023;9(1):30. doi: 10.1038/s41421-022-00513-z 36928215 PMC10020559

[34] Wu Y, Su K, Zhang Y, Liang L, Wang F, Chen S. A spatiotemporal transcriptomic atlas of mouse placentation. Cell Discov. 2024;10(1):110. 39438452 10.1038/s41421-024-00740-6 PMC11496649

[35] Benoit C, Gu Y, Zhang Y, Alexander JS, Wang Y. Contractility of placental vascular smooth muscle cells in response to stimuli produced by the placenta: roles of ACE vs. non-ACE and AT1 vs. AT2 in placental vessel cells. Placenta. 2008;29(6):503–9. doi: 10.1016/j.placenta.2008.03.002 18417209 PMC2556283

[36] Pollheimer J, Vondra S, Baltayeva J, Beristain AG, Knöfler M. Regulation of placental extravillous trophoblasts by the maternal uterine environment. Front Immunol. 2018;9:2597. doi: 10.3389/fimmu.2018.02597 30483261 PMC6243063

[37] Abbott NJ, Rönnbäck L, Hansson E. Astrocyte–endothelial interactions at the blood–brain barrier. Nat Rev Neurosci. 2006;7(1):41–53. 16371949 10.1038/nrn1824

[38] Durkee CA, Araque A. Diversity and specificity of astrocyte-neuron communication. Neuroscience. 2019;396:73–8. doi: 10.1016/j.neuroscience.2018.11.010 30458223 PMC6494094

[39] Jha MK, Jo M, Kim J-H, Suk K. Microglia-astrocyte crosstalk: an intimate molecular conversation. Neuroscientist. 2019;25(3):227–40. doi: 10.1177/1073858418783959 29931997

[40] Nutma E, van Gent D, Amor S, Peferoen LAN. Astrocyte and oligodendrocyte cross-talk in the central nervous system. Cells. 2020;9(3):600. doi: 10.3390/cells9030600 32138223 PMC7140446

[41] Grammatopoulos DK, Ourailidou S. CRH receptor signalling: potential roles in pathophysiology. Curr Mol Pharmacol. 2017;10(4):296–310. doi: 10.2174/1874467210666170110125747 28103786

[42] Maruyama H, Makino S, Noguchi T, Nishioka T, Hashimoto K. Central type 2 corticotropin-releasing hormone receptor mediates hypothalamic-pituitary-adrenocortical axis activation in the rat. Neuroendocrinology. 2007;86(1):1–16. doi: 10.1159/000103556 17551262

[43] Zheng Y, Zhang Y-M, Ni X. Urocortin 2 but not urocortin 3 promotes the synaptic formation in hipppocampal neurons via induction of NGF production by astrocytes. Endocrinology. 2016;157(3):1200–10. doi: 10.1210/en.2015-1812 26713785

[44] Hunyady Á, Hajna Z, Gubányi T, Scheich B, Kemény Á, Gaszner B, et al. Hemokinin-1 is an important mediator of pain in mouse models of neuropathic and inflammatory mechanisms. Brain Res Bull. 2019;147:165–73. doi: 10.1016/j.brainresbull.2019.01.015 30664920

[45] Yu H, Rubinstein M, Low MJ. Developmental single-cell transcriptomics of hypothalamic POMC neurons reveal the genetic trajectories of multiple neuropeptidergic phenotypes. Elife. 2022;11:e72883. 10.7554/eLife.72883 PMC8806186 35044906

[46] Herb BR, Glover HJ, Bhaduri A, Colantuoni C, Bale TL, Siletti K, et al. Single-cell genomics reveals region-specific developmental trajectories underlying neuronal diversity in the human hypothalamus. Sci Adv. 2023;9(45):eadf6251. 10.1126/sciadv.adf6251 PMC10631741 37939194

[47] Kaplan HS, Logeman BL, Zhang K, Santiago C, Sohail N, Naumenko S, et al. Sensory input, sex, and function shape hypothalamic cell type development. bioRxiv. 2024:2024.01.23.576835. doi: 10.1101/2024.01.23.576835 38328205 PMC10849564

[48] Zhou X, Lu Y, Zhao F, Dong J, Ma W, Zhong S, et al. Deciphering the spatial-temporal transcriptional landscape of human hypothalamus development. Cell Stem Cell. 2022;29(2):328-343.e5. doi: 10.1016/j.stem.2021.11.009 34879244

[49] Gullotta GS, Costantino G, Sortino MA, Spampinato SF. Microglia and the blood-brain barrier: an external player in acute and chronic neuroinflammatory conditions. Int J Mol Sci. 2023;24(11):9144. doi: 10.3390/ijms24119144 37298096 PMC10252410

[50] Paredes MF, Mora C, Flores-Ramirez Q, Cebrian-Silla A, Del Dosso A, Larimer P, et al. Nests of dividing neuroblasts sustain interneuron production for the developing human brain. Science. 2022;375(6579):eabk2346. doi: 10.1126/science.abk2346 35084970 PMC8887556

[51] Wang H, Xu L, Lai C, Hou K, Chen J, Guo Y. Region-specific distribution of Olig2-expressing astrocytes in adult mouse brain and spinal cord. Mol Brain. 2021;14:1–14. 33618751 10.1186/s13041-021-00747-0 PMC7901088

[52] Coutu DL, Galipeau J. Roles of FGF signaling in stem cell self-renewal, senescence and aging. Aging (Albany NY). 2011;3(10):920–33. doi: 10.18632/aging.100369 21990129 PMC3229969

[53] Oike Y, Yasunaga K, Suda T. Angiopoietin-related/angiopoietin-like proteins regulate angiogenesis. Int J Hematol. 2004;80:21–8. 15293564 10.1532/ijh97.04034

[54] Santulli G. Angiopoietin-like proteins: a comprehensive look. Front Endocrinol (Lausanne). 2014;5:4. doi: 10.3389/fendo.2014.00004 24478758 PMC3899539

[55] Di Bella DJ, Habibi E, Stickels RR, Scalia G, Brown J, Yadollahpour P, et al. Molecular logic of cellular diversification in the mouse cerebral cortex. Nature. 2021;595(7868):554–9. doi: 10.1038/s41586-021-03670-5 34163074 PMC9006333

[56] Pollen AA, Nowakowski TJ, Shuga J, Wang X, Leyrat AA, Lui JH, et al. Low-coverage single-cell mRNA sequencing reveals cellular heterogeneity and activated signaling pathways in developing cerebral cortex. Nat Biotechnol. 2014;32(10):1053–8. doi: 10.1038/nbt.2967 25086649 PMC4191988

[57] Di Virgilio F, Vuerich M. Purinergic signaling in the immune system. Auton Neurosci. 2015;191:117–23. doi: 10.1016/j.autneu.2015.04.011 25979766

[58] Falomir-Lockhart LJ, Cavazzutti GF, Giménez E, Toscani AM. Fatty acid signaling mechanisms in neural cells: fatty acid receptors. Front Cell Neurosci. 2019;13:162. doi: 10.3389/fncel.2019.00162 31105530 PMC6491900

[59] Wang X, Lou N, Xu Q, Tian G-F, Peng WG, Han X, et al. Astrocytic Ca2+ signaling evoked by sensory stimulation in vivo. Nat Neurosci. 2006;9(6):816–23. doi: 10.1038/nn1703 16699507

[60] Kumar P, Ohana O. Inter- and intralaminar subcircuits of excitatory and inhibitory neurons in layer 6a of the rat barrel cortex. J Neurophysiol. 2008;100(4):1909–22. doi: 10.1152/jn.90684.2008 18650305

[61] Feldmeyer D. Structure and function of neocortical layer 6b. Front Cell Neurosci. 2023;17:1257803. doi: 10.3389/fncel.2023.1257803 37744882 PMC10516558

[62] Munyeshyaka M, Fields RD. Oligodendroglia are emerging players in several forms of learning and memory. Commun Biol. 2022;5(1):1148. doi: 10.1038/s42003-022-04116-y 36309567 PMC9617857

[63] Yi JJ, Barnes AP, Hand R, Polleux F, Ehlers MD. TGF-beta signaling specifies axons during brain development. Cell. 2010;142(1):144–57. doi: 10.1016/j.cell.2010.06.010 20603020 PMC2933408

[64] Manzari-Tavakoli A, Babajani A, Farjoo MH, Hajinasrollah M, Bahrami S, Niknejad H. The cross-talks among Bone Morphogenetic Protein (BMP) signaling and other prominent pathways involved in neural differentiation. Front Mol Neurosci. 2022;15:827275. doi: 10.3389/fnmol.2022.827275 35370542 PMC8965007

[65] Hirano S, Suzuki ST, Redies C. The cadherin superfamily in neural development: diversity, function and interaction with other molecules. Front Biosci. 2003;8:d306-55. doi: 10.2741/972 12456358

[66] Larsen M, Tremblay ML, Yamada KM. Phosphatases in cell-matrix adhesion and migration. Nat Rev Mol Cell Biol. 2003;4(9):700–11. doi: 10.1038/nrm1199 14506473

[67] den Haan JMM, Arens R, van Zelm MC. The activation of the adaptive immune system: cross-talk between antigen-presenting cells, T cells and B cells. Immunol Lett. 2014;162(2 Pt B):103–12. doi: 10.1016/j.imlet.2014.10.011 25455596

[68] Hughes CCW. Endothelial-stromal interactions in angiogenesis. Curr Opin Hematol. 2008;15(3):204–9. doi: 10.1097/MOH.0b013e3282f97dbc 18391786 PMC6606556

[69] Liu L, Michowski W, Kolodziejczyk A, Sicinski P. The cell cycle in stem cell proliferation, pluripotency and differentiation. Nat Cell Biol. 2019;21(9):1060–7. doi: 10.1038/s41556-019-0384-4 31481793 PMC7065707

[70] Liu Y, Shen X, Zhang Y, Zheng X, Cepeda C, Wang Y, et al. Interactions of glial cells with neuronal synapses, from astrocytes to microglia and oligodendrocyte lineage cells. Glia. 2023;71(6):1383–401. doi: 10.1002/glia.24343 36799296

[71] Parikh K, Antanaviciute A, Fawkner-Corbett D, Jagielowicz M, Aulicino A, Lagerholm C, et al. Colonic epithelial cell diversity in health and inflammatory bowel disease. Nature. 2019;567(7746):49–55. doi: 10.1038/s41586-019-0992-y 30814735

[72] Cayrol C, Girard JP. Interleukin-33 (IL-33): a nuclear cytokine from the IL-1 family. Immunol Rev. 2018;281(1):154–68. 29247993 10.1111/imr.12619

[73] Elmentaite R, Kumasaka N, Roberts K, Fleming A, Dann E, King HW, et al. Cells of the human intestinal tract mapped across space and time. Nature. 2021;597(7875):250–5. doi: 10.1038/s41586-021-03852-1 34497389 PMC8426186

[74] Xu Q, Hou W, Zhao B, Fan P, Wang S, Wang L, et al. Mesenchymal stem cells lineage and their role in disease development. Mol Med. 2024;30(1):207. doi: 10.1186/s10020-024-00967-9 39523306 PMC11552129

