# Supplementary material for: OrgaCCC: Orthogonal graph autoencoders for constructing cell-cell communication networks on spatial transcriptomics data
Source: PLoS Comput Biol. 2025 Jun 27;21(6):e1013212. doi: 10.1371/journal.pcbi.1013212 (PMC12258598; doi:10.1371/journal.pcbi.1013212)
Supplement: S1 Fig — a, Comparison of cellular communication predicted by OrgaCCC (left) and directly obtained from the original cell spatial graph (right). Colors represent the communication strength, with red indicating stronger interactions and blue indicating weaker interactions. b, Simulation of missing edges in cell spatial graph. Randomly remove different proportions of real edges in the cell spatial graph, bring them into the model training to get the AUC value, repeat the process thirty times and plot the boxplots. c, Cell types mainly contained in each cluster obtained by spectral clustering of cell graph A^c. d, The overlap of ligand-receptor pairs predicted by CellChat with COMMOT, iTALK, NiCo and OrgaCCC, by iTALK with CellChat, OrgaCCC, NiCo and COMMOT, and by NiCo with CellChat, OrgaCCC, iTALK and COMMOT. (PDF) [file pcbi.1013212.s001.pdf]

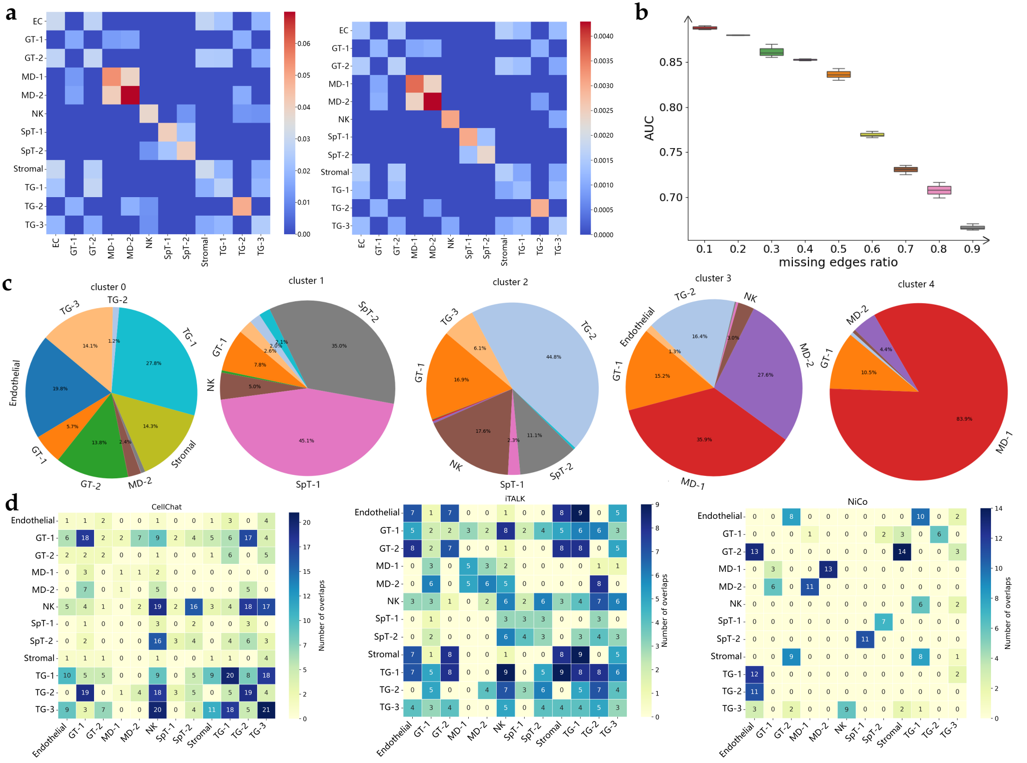

S1 Fig. Downstream analysis on STARmap data of the mouse placenta. a, Comparison of cellular communication predicted by OrgaCCC (left) and directly obtained from the original cell spatial graph (right). Colors represent the communication strength, with red indicating stronger interactions and blue indicating weaker interactions. **b**, Simulation of missing edges in cell spatial graph. Randomly remove different proportions of real edges in the cell spatial graph, bring them into the model training to get the AUC value, repeat the process thirty times and plot the boxplots. **c**, Cell types mainly contained in each cluster obtained by spectral clustering of cell graph $\hat{A}_c$. **d**, The overlap of ligand-receptor pairs predicted by CellChat with COMMOT, iTALK, NiCo and OrgaCCC, by iTALK with CellChat, OrgaCCC, NiCo and COMMOT, and by NiCo with CellChat, OrgaCCC, iTALK and COMMOT.
