# Supplementary material for: OrgaCCC: Orthogonal graph autoencoders for constructing cell-cell communication networks on spatial transcriptomics data
Source: PLoS Comput Biol. 2025 Jun 27;21(6):e1013212. doi: 10.1371/journal.pcbi.1013212 (PMC12258598; doi:10.1371/journal.pcbi.1013212)
Supplement: S2 Fig — a, Comparison of cellular communication predicted by OrgaCCC (left) and the original cell spatial graph (right). b, Predicting the number of cell pairs with intercellular communication relationships at different distances by OrgaCCC, DeepLinc and COMMOT. c, Cell types in each cluster from spectral clustering of cell graph A^c. d, The overlap of ligand-receptor pairs predicted by CellChat with COMMOT, iTALK, NiCo and OrgaCCC, by iTALK with CellChat, OrgaCCC, NiCo and COMMOT, and by NiCo with CellChat, OrgaCCC, iTALK and COMMOT. e, The top eight biological processes obtained by enrichment analysis using the top sensitive partial genes. f, Simulation of missing edges and add fake edges in cell spatial graph. Randomly remove different proportions of real edges or add fake edges in the cell spatial graph, bring them into the model training to get the AUC value, repeat the process thirty times and plot the boxplots. (PDF) [file pcbi.1013212.s002.pdf]

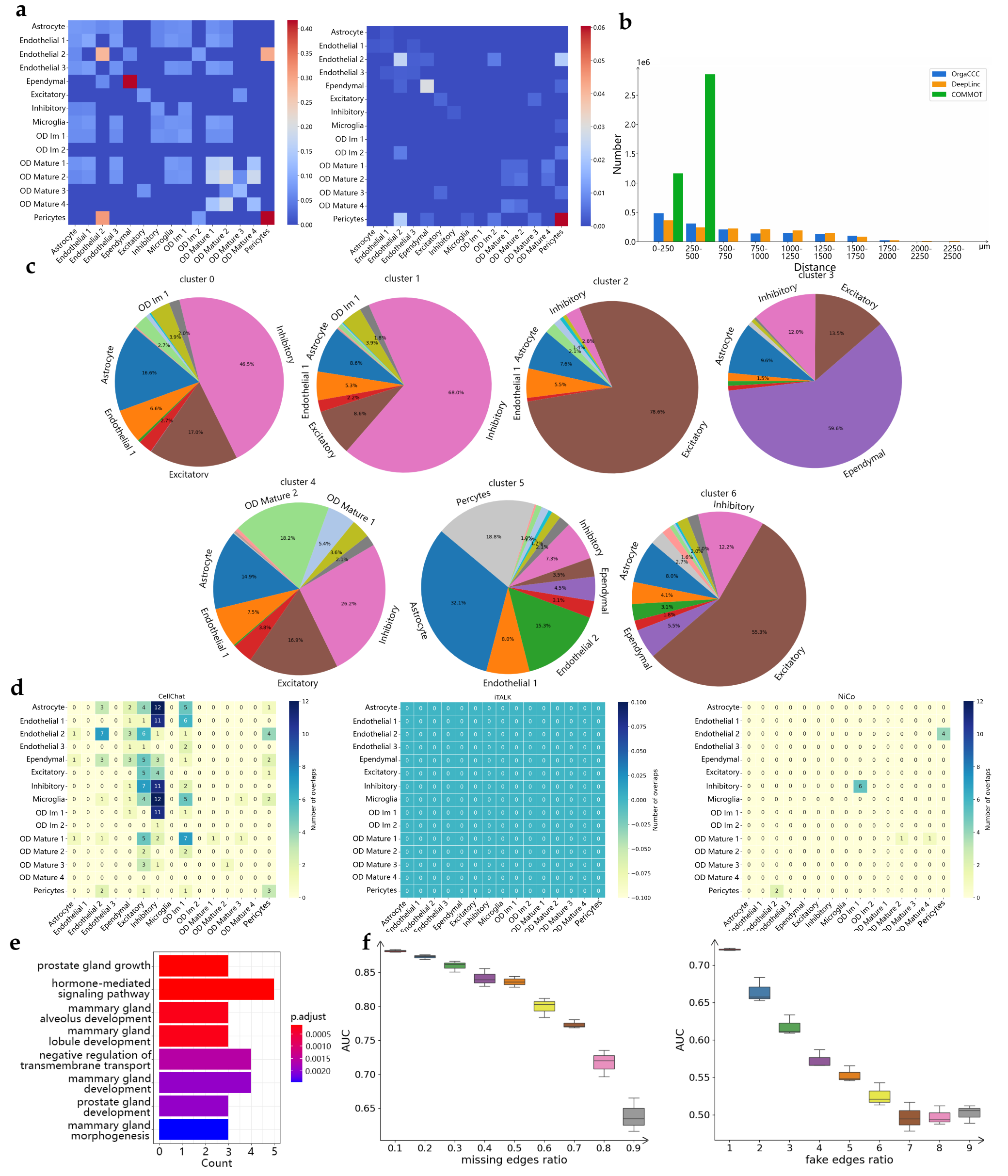

S2 Fig. Downstream analysis on MERFISH data of mouse hypothalamic preoptic region. **a**, Comparison of cellular communication predicted by OrgaCCC (left) and the original cell spatial graph (right). **b**, Predicting the number of cell pairs with intercellular communication relationships at different distances by OrgaCCC, DeepLinc and COMMOT. **c**, Cell types in each cluster from spectral clustering of cell graph $\hat{A}_c$. **d**, The overlap of ligand-receptor pairs predicted by CellChat with COMMOT, iTALK, NiCo and OrgaCCC, by iTALK with CellChat, OrgaCCC, NiCo and COMMOT, and by NiCo with CellChat, OrgaCCC, iTALK and COMMOT. **e**, The top eight biological processes obtained by enrichment analysis using the top sensitive partial genes. **f**, Simulation of missing edges and add fake edges in cell spatial graph. Randomly remove different proportions of real edges or add fake edges in the cell spatial graph, bring them into the model training to get the AUC value, repeat the process thirty times and plot the boxplots.
