# Supplementary material for: OrgaCCC: Orthogonal graph autoencoders for constructing cell-cell communication networks on spatial transcriptomics data
Source: PLoS Comput Biol. 2025 Jun 27;21(6):e1013212. doi: 10.1371/journal.pcbi.1013212 (PMC12258598; doi:10.1371/journal.pcbi.1013212)
Supplement: S3 Fig — a, Celltype-level of OrgaCCC, CellChat, COMMOT, iTALK prediction of the overlap of results. Numbers represent the pairs of predictions that overlap at the celltype-level. b, The overlap of ligand-receptor pairs predicted by CellChat (top) with COMMOT, iTALK, and OrgaCCC, and by iTALK (bottom) with CellChat, OrgaCCC, and COMMOT. c, Cell types mainly contained in each cluster obtained by spectral clustering of cell graph A^c. d, Predicting the number of cell pairs with intercellular communication relationships at different distances by OrgaCCC, DeepLinc and COMMOT. e, Simulation of missing edges and add fake edges in cell spatial graph. Randomly remove different proportions of real edges or add fake edges in the cell spatial graph, bring them into the model training to get the AUC value, repeat the process thirty times and plot the boxplots. (PDF) [file pcbi.1013212.s003.pdf]

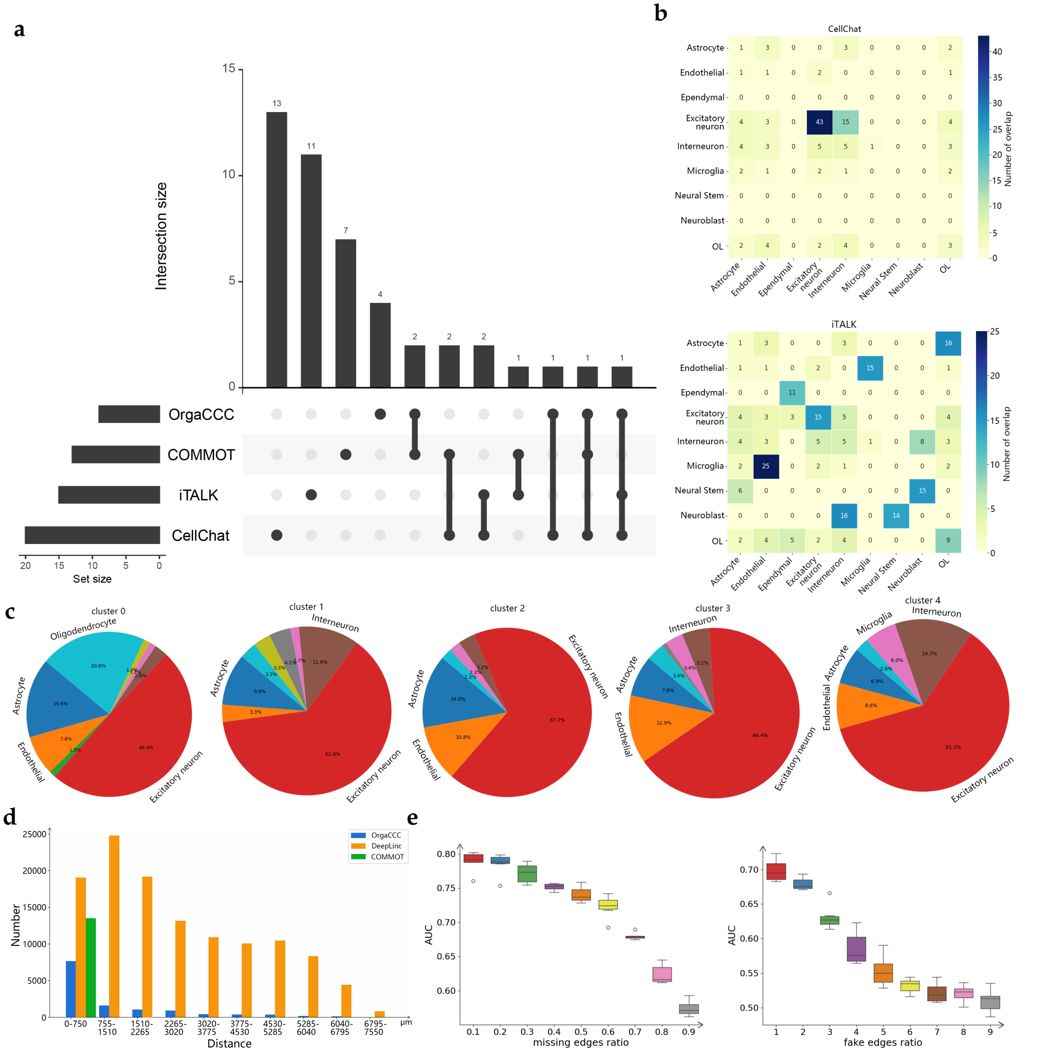

S3 Fig. Downstream analysis on seqFISH+ data of mouse secondary somatosensory cortex. **a**, Celltype-level of OrgaCCC, CellChat, COMMOT, iTALK prediction of the overlap of results. Numbers represent the pairs of predictions that overlap at the celltype-level. **b**, The overlap of ligand-receptor pairs predicted by CellChat (top) with COMMOT, iTALK, and OrgaCCC, and by iTALK (bottom) with CellChat, OrgaCCC, and COMMOT. **c**, Cell types mainly contained in each cluster obtained by spectral clustering of cell graph $\hat{A}_c$. **d**, Predicting the number of cell pairs with intercellular communication relationships at different distances by OrgaCCC, DeepLinc and COMMOT. **e**, Simulation of missing edges and add fake edges in cell spatial graph. Randomly remove different proportions of real edges or add fake edges in the cell spatial graph, bring them into the model training to get the AUC value, repeat the process thirty times and plot the boxplots.
