# Supplementary figures and images for: OrgaCCC: Orthogonal graph autoencoders for constructing cell-cell communication networks on spatial transcriptomics data
Source: PLoS Comput Biol. 2025 Jun 27;21(6):e1013212. doi: 10.1371/journal.pcbi.1013212 (PMC12258598; doi:10.1371/journal.pcbi.1013212)

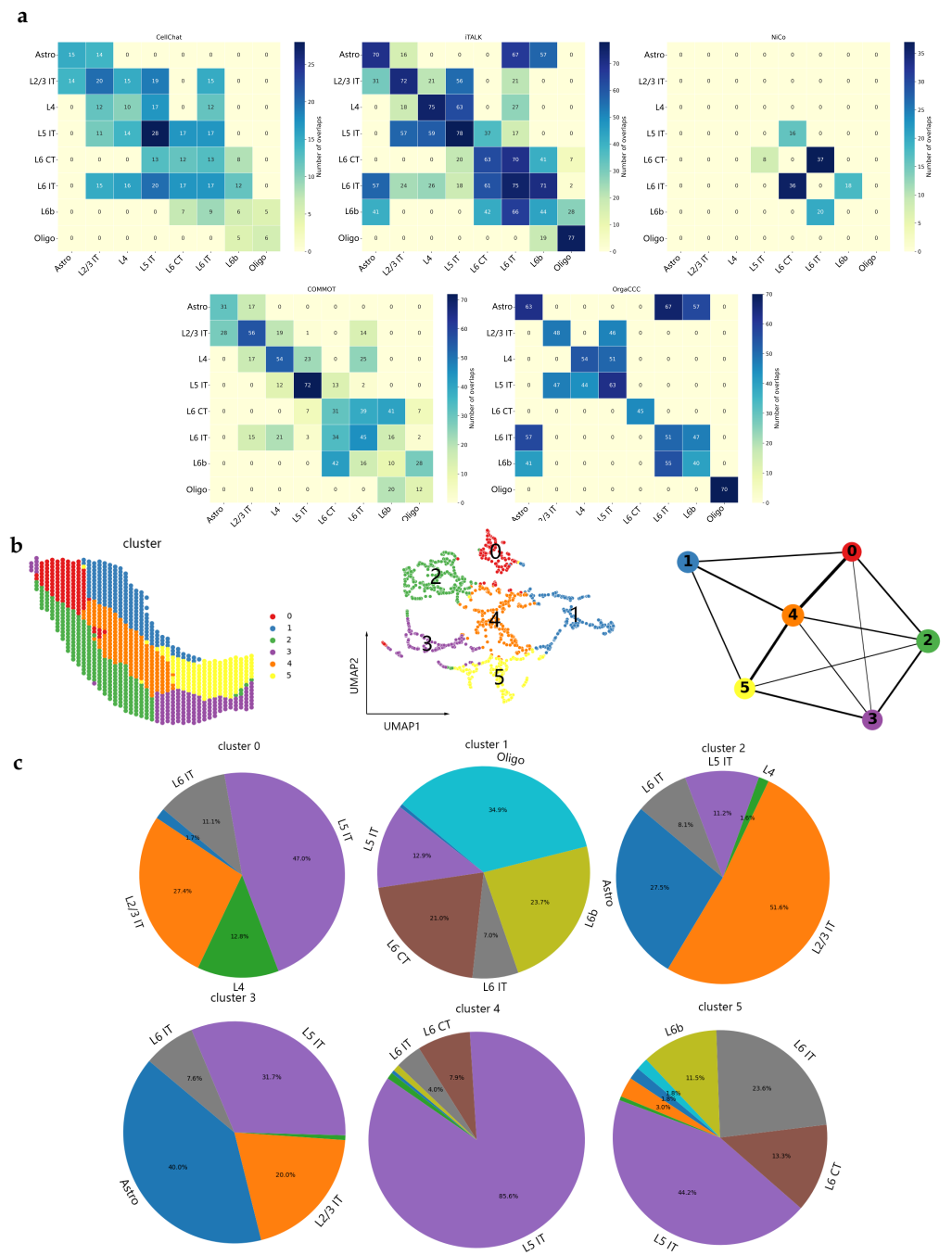

Supplement: S4 Fig — a, The overlap of ligand-receptor pairs predicted by CellChat with NiCo, COMMOT, iTALK, and OrgaCCC, by iTALK with CellChat, NiCo, OrgaCCC, and COMMOT, by NiCo with CellChat, COMMOT, OrgaCCC, and iTALK, by COMMOT with CellChat, NiCo, OrgaCCC, and iTALK, and by OrgaCCC with CellChat, COMMOT, NiCo, and iTALK, respectively. b, The cell clustering results obtained by spectral clustering of cell graph A^c, and the corresponding UMAP and PAGA graphs. c, Cell types mainly contained in each cluster obtained by spectral clustering of cell graph A^c. (PDF) [file pcbi.1013212.s004.pdf]
