# Supplementary material for: OrgaCCC: Orthogonal graph autoencoders for constructing cell-cell communication networks on spatial transcriptomics data
Source: PLoS Comput Biol. 2025 Jun 27;21(6):e1013212. doi: 10.1371/journal.pcbi.1013212 (PMC12258598; doi:10.1371/journal.pcbi.1013212)
Supplement: S5 Fig — a, The overlap of ligand-receptor pairs predicted by CellChat with NiCo, COMMOT, iTALK, and OrgaCCC, by iTALK with CellChat, NiCo, OrgaCCC, and COMMOT, by NiCo with CellChat, COMMOT, OrgaCCC, and iTALK, by COMMOT with CellChat, NiCo, OrgaCCC, and iTALK, and by OrgaCCC with CellChat, COMMOT, NiCo, and iTALK, respectively. b, The Cell clustering related UMAP and PAGA graphs. c, The top eight biological processes obtained by enrichment analysis using the top sensitive partial genes. d, Cell types mainly contained in each cluster obtained by spectral clustering of cell graph A^c. (PDF) [file pcbi.1013212.s005.pdf]

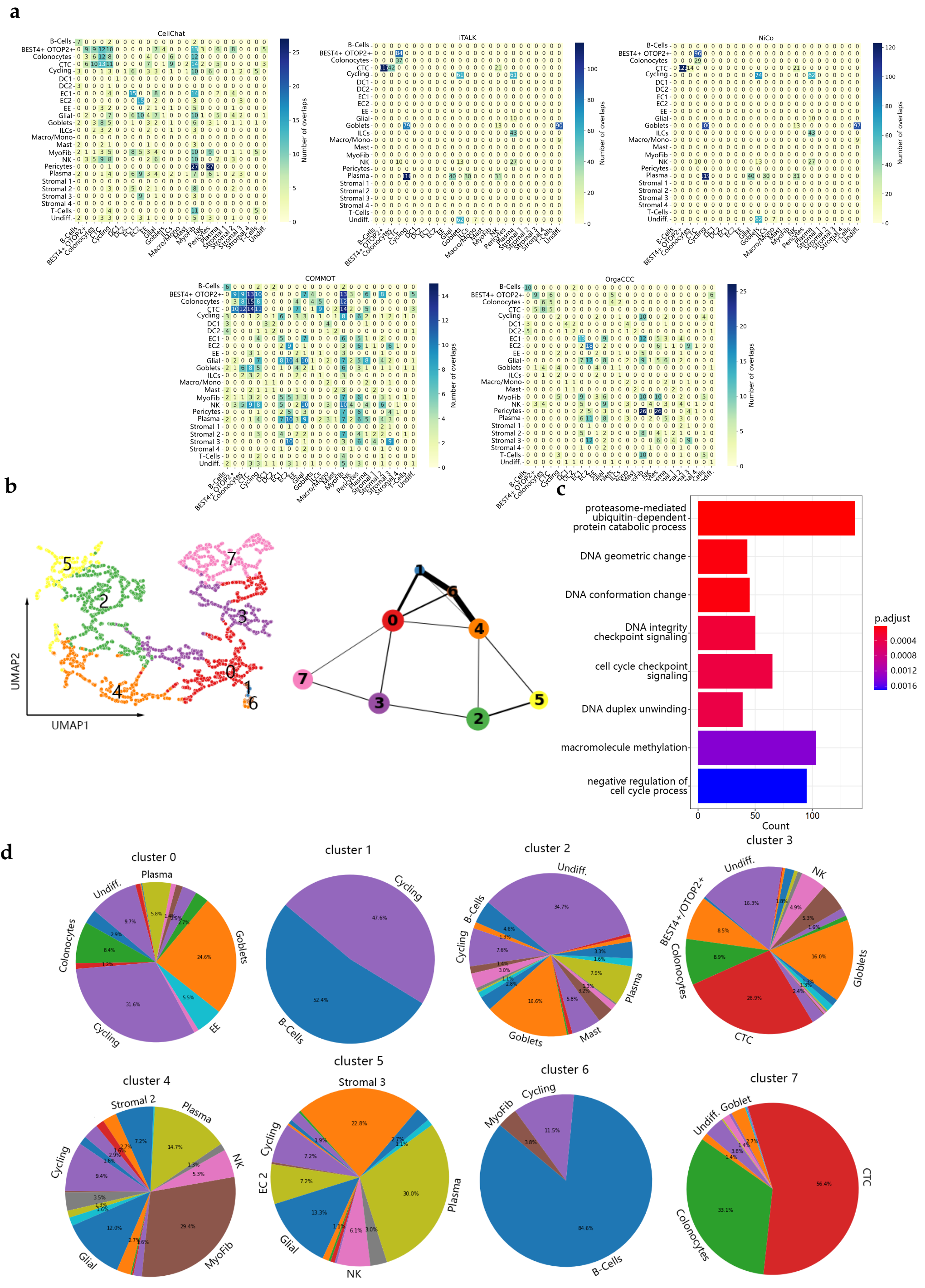

S5 Fig. Downstream analysis on seqFISH+ data of human intestine. **a**, The overlap of ligand-receptor pairs predicted by CellChat with NiCo, COMMOT, iTALK, and OrgaCCC, by iTALK with CellChat, NiCo, OrgaCCC, and COMMOT, by NiCo with CellChat, COMMOT, OrgaCCC, and iTALK, by COMMOT with CellChat, NiCo, OrgaCCC, and iTALK, and by OrgaCCC with CellChat, COMMOT, NiCo, and iTALK, respectively. **b**, The Cell clustering related UMAP and PAGA graphs. **c**, The top eight biological processes obtained by enrichment analysis using the top sensitive partial genes. **d**, Cell types mainly contained in each cluster obtained by spectral clustering of cell graph $\hat{A}_c$.
